# Whole-genome reconstruction and mutational signatures in gastric cancer

**DOI:** 10.1186/gb-2012-13-12-r115

**Published:** 2012-12-13

**Authors:** Niranjan Nagarajan, Denis Bertrand, Axel M Hillmer, Zhi Jiang Zang, Fei Yao, Pierre-Étienne Jacques, Audrey SM Teo, Ioana Cutcutache, Zhenshui Zhang, Wah Heng Lee, Yee Yen Sia, Song Gao, Pramila N Ariyaratne, Andrea Ho, Xing Yi Woo, Lavanya Veeravali, Choon Kiat Ong, Niantao Deng, Kartiki V Desai, Chiea Chuen Khor, Martin L Hibberd, Atif Shahab, Jaideepraj Rao, Mengchu Wu, Ming Teh, Feng Zhu, Sze Yung Chin, Brendan Pang, Jimmy BY So, Guillaume Bourque, Richie Soong, Wing-Kin Sung, Bin Tean Teh, Steven Rozen, Xiaoan Ruan, Khay Guan Yeoh, Patrick BO Tan, Yijun Ruan

**Affiliations:** 1 Computational and Systems Biology, Genome Institute of Singapore, Singapore 138672, Singapore; 2 Genome Technology and Biology, Genome Institute of Singapore, Singapore 138672, Singapore; 3 Cellular and Molecular Research, National Cancer Centre, Singapore 169610, Singapore; 4 Cancer and Stem Cell Biology Program, Duke-National University of Singapore (NUS) Graduate Medical School, Singapore 169857, Singapore; 5 Department of Epidemiology and Public Health, Yong Loo Lin School of Medicine, National University of Singapore, Singapore 119074, Singapore; 6 Neuroscience and Behavioral Disorders, Duke-NUS Graduate Medical School, Singapore 169857, Singapore; 7 NUS Graduate School of Integrative Sciences and Engineering, Centre for Life Sciences, Singapore 117456, Singapore; 8 Research Computing, Genome Institute of Singapore, Singapore 138672, Singapore; 9 NCCS-VARI Translational Research Laboratory, National Cancer Centre, Singapore 169610, Singapore; 10 Genomic Oncology, Duke-NUS Graduate Medical School, Singapore 169857, Singapore; 11 National Institute of Biomedical Genomics, 2nd Floor Netaji Subash Sanatorium, Kalyani 741251 West Bengal, India; 12 Infectious Diseases, Genome Institute of Singapore, Singapore 138672, Singapore; 13 Department of Surgery, Tan Tock Seng Hospital, Singapore 308433, Singapore; 14 Cancer Science Institute of Singapore, Yong Loo Lin School of Medicine, National University of Singapore, Singapore 119074, Singapore; 15 Department of Pathology, National University Health System, National University of Singapore, Singapore 119074, Singapore; 16 Department of Medicine, National University Health System, National University of Singapore, Singapore 119074, Singapore; 17 Department of Surgery, National University Health System, National University of Singapore, Singapore 119074, Singapore; 18 Department of Human Genetics, McGill University, Montréal H3A 1B, Canada; 19 McGill University and Genome Quebec Innovation Center, Montréal H3A 1A4, Canada; 20 Department of Biochemistry, National University of Singapore, Singapore 119074, Singapore

## Abstract

**Background:**

Gastric cancer is the second highest cause of global cancer mortality. To explore the complete repertoire of somatic alterations in gastric cancer, we combined massively parallel short read and DNA paired-end tag sequencing to present the first whole-genome analysis of two gastric adenocarcinomas, one with chromosomal instability and the other with microsatellite instability.

**Results:**

Integrative analysis and *de novo *assemblies revealed the architecture of a wild-type *KRAS *amplification, a common driver event in gastric cancer. We discovered three distinct mutational signatures in gastric cancer - against a genome-wide backdrop of oxidative and microsatellite instability-related mutational signatures, we identified the first exome-specific mutational signature. Further characterization of the impact of these signatures by combining sequencing data from 40 complete gastric cancer exomes and targeted screening of an additional 94 independent gastric tumors uncovered *ACVR2A*, *RPL22 *and *LMAN1 *as recurrently mutated genes in microsatellite instability-positive gastric cancer and *PAPPA *as a recurrently mutated gene in *TP53 *wild-type gastric cancer.

**Conclusions:**

These results highlight how whole-genome cancer sequencing can uncover information relevant to tissue-specific carcinogenesis that would otherwise be missed from exome-sequencing data.

## Background

Gastric cancer (GC) is the fourth most common cancer and the second leading cause of cancer death worldwide. Early stage GC is often asymptomatic or associated with non-specific symptoms, resulting in most patients presenting at advanced disease stages. Treatment options for late-stage GC patients are limited, with surgery and chemotherapy regimens offering modest survival benefits. Environmental risk factors for GC include a high salt diet, smoking, and infection by *Helicobacter pylori *[1]. Understanding the mutational impact of these environmental exposures on the genomes of gastric epithelial cells is essential to shed light on specific genes and pathways associated with gastric tumorigenesis.

Previous studies in lung cancer [2,3], melanoma [4], and leukemia [5] have shown that environmental carcinogens and drugs can elicit specific somatic mutational profiles in cancer genomes, referred to as 'mutational signatures'. While previous studies on GC have applied exome-sequencing approaches to identify frequently mutated genes [6,7], identifying mutational signatures is best done using whole-genome data, due to its completeness and ability to simultaneously uncover micro- and macro-scale somatic alterations. In this study, we sought to provide a more comprehensive understanding of mutational processes in GC by analyzing whole-genome sequences of two GCs and their matched-normal controls, using both short-read (SR) next-generation sequencing and a long insert (approximately 10 kbp) DNA paired-end tag (DNA-PET) protocol [8]. We also sought to explore the combination of these datasets for *de novo *assembly of cancer and normal genomes and to comprehensively catalogue a range of (point mutations to megabase-sized) somatic alterations in the tumor. Finally, we used this catalogue to characterize the impact of mutational processes on genes and used a screening approach to validate recurrently mutated genes in subtypes of GC defined by specific mutational processes.

## Results

### Integrative short read/DNA-PET analysis and *de novo *assembly

The matched tumor and normal samples analyzed were from two Singaporean patients. One GC exhibited evidence of microsatellite instability (MSI) and active *H. pylori *infection (see Table S1 in Additional file 1 for other clinical characteristics). Each tumor and matched normal sample was sequenced to more than 30-fold average base pair coverage by Illumina SR sequencing (Materials and methods; Table S2 in Additional file 1), and to > 130-fold physical coverage using large-insert (approximately 10 kbp) DNA-PET sequencing [9] on the SOLiD platform (Materials and methods; Table S3 and Note 1 in Additional file 1). Single nucleotide variants (SNVs) and short insertions and deletions (indels) from tumor and normal genomes were combined to identify somatic variants (Table 1 and Materials and methods) and reliability of somatic calls was confirmed using targeted sequencing (validation rate of 90% for SNVs and 96% for indels; Materials and methods). SR and DNA-PET data were also used to identify somatic copy-number variations (CNVs) and structural variations (SVs) (validation rate = 81%; Materials and methods; Note 1 in Additional file 1).

**Table 1:** Somatic variations in two GC tumors identified by whole genome sequencing approaches

Patient ID	NGCII082	NGCII092
SNVs, all somatic	14,856	17,473
Coding regions	119	116
Non-synonymous	86	73
Promoter regions	101	161
Indels, all somatic	11,738	2,486
Coding regions	12	2
CNVs, all somatic	836	21,776
Affecting genes	3	265
SVs, all somatic	12	146
Affecting genes	11	96
Deletions	6	56
Tandem duplications	2	8
Unpaired inversions	0	26
Inversions	0	2
Insertions (intra-chromosomal)	0	0
Insertions (inter-chromosomal)	0	0
Isolated translocations	0	3
Balanced translocations	0	0
Complex events (intra- chromosomal)	4	49
Complex events (inter- chromosomal)	0	2

**Figure 1:**
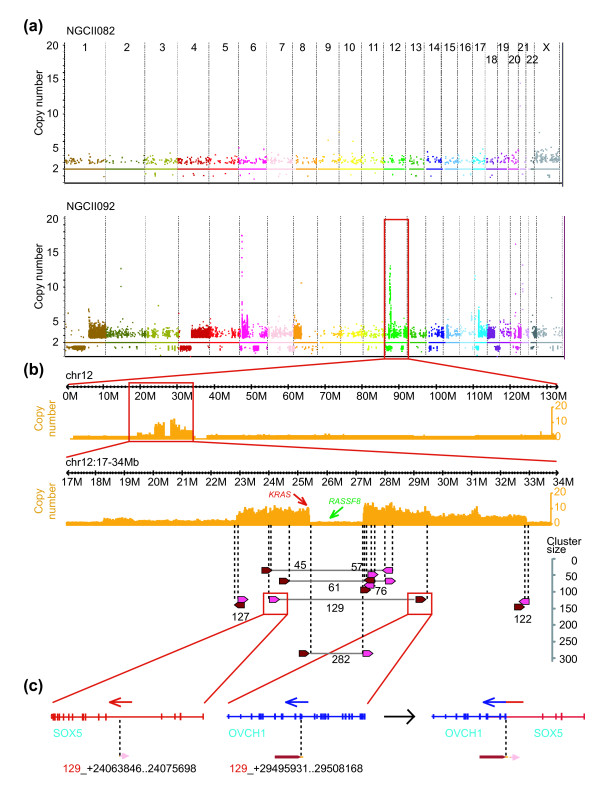
**Copy number of two gastric cancer genomes, mechanism of 12p amplification and creation of a fusion gene**. **(a) **Somatic CNVs in the two gastric tumors (chromosomes are arranged on the x-axis, copy number is shown on the y-axis). **(b) **Copy number of chromosome 12 (top) and the amplicon on 12p (middle) are shown in orange (y-axis). Rearrangements identified by DNA-PET clusters with a size ≥ 45 are represented by arrows and connecting lines (bottom). Dark red and pink arrows represent 5' and 3' cluster regions, respectively, with the connection between the tip of the dark red and the blunt end of the pink arrows. Numbers represent cluster sizes. **(c) **Fusion between *SOX5 *and *OVCH1 *predicted by a rearrangement point with cluster size of 129 in (b).

We integrated the SR and DNA-PET sequence information to perform *de novo *assembly of the tumor and normal genomes. While complete *de novo *assembly of a tumor genome still poses significant technical challenges and has not been attempted before, we were able to use the SR/DNA-PET data to construct highly contiguous draft assemblies of median scaffold lengths (N50) in the range of 41 to 148 kb, with DNA-PET data assisting in tripling sequence contiguity of the assemblies (Materials and methods; Note 2 and Table S5 in Additional file 1). Importantly, performing *de novo *SR/DNA-PET assembly revealed several findings not observed using conventional analyses of the SR data. First, the *de novo *approach allowed for characterization of large-scale somatic structural variations at single base-pair resolution (SR libraries were unable to identify nearly half of the validated SVs and fusions genes; Note 1 in Additional file 1). For example, NGCII092 exhibited a focal genomic amplification on chromosome 12p11-12 in a region containing the wild-type *KRAS *gene, a genomic event frequently observed in GC [10]. The combined SR/DNA-PET data (Materials and methods) enabled a detailed putative reconstruction of the evolutionary lineage of the amplified *KRAS *locus with concomitant deletion of a proposed tumor suppressor gene *RASSF8 *(as well as another focal amplicon at chromosome 6p) as described in the supplementary text (Figure 1; Figures S1 and S2 and Note 3 in Additional file 1). Reconstruction of the tumor genomes also allowed the prediction of fusion genes and complex rearrangements that resemble patterns created by replication coupled mechanisms [11] and are further described in the supplementary text (Note 4 and Figures S3 and S4 in Additional file 1 and Table S6 in Additional file 2).

Second, a combined SR/DNA-PET analysis allowed us to assemble sequences present in the tumor genome but not in the reference human genome. For example, in patient NGCII082 exhibiting active *H. pylori *infection, we detected approximately 2,000 short-sequence reads and > 600 DNA-PET tags corresponding to the *H. pylori *genome (the first such report for a bacterial pathogen from tumor sequencing), in addition to a tumor-associated microbiome (these were not seen in NGCII092; see Figure S5 and Note 5 in Additional file 1 for details). Note that, despite being fewer in number, the DNA-PET tags contributed significantly to the physical coverage and analysis of the genomes (Figure S5 and Note 5 in Additional file 1).

Third, the *de novo *assembly enabled annotation of human genes and variants in sequences absent in the reference genome. In total, we identified more than 3 Mbp of novel sequence (longer than 500 bp), containing several genes (including an ortholog to a cytokine receptor-like factor - *CRLF2*), and more than a 1,000 somatic and germline variants for each patient (Materials and methods; Note 2 and Table S5 in Additional file 1).

### Mutational signatures of damage by reactive oxygen species, deamination and microsatellite instability

**Figure 2:**
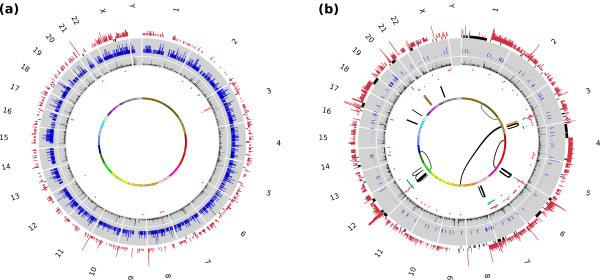
**Map of somatic alterations in two gastric cancer genomes**. The Circos plots depict the following information in order from outer to inner rings: using WGS data (1) CNVs (gain in red capped at 10 copies and loss in gray), (2) indel density (indel frequency per 10 kbp in blue, capped at 5 indels/10 kbp), (3) SNV density (SNV frequency per 10 kbp in black, each ring is 5 SNVs/10 kbp, capped at 10), and using DNA-PET data, (4) deletions (in red), tandem duplications (green) and inversions (purple), (5) intra- and (6) inter-chromosomal, insertions (orange) and unpaired SVs (gray).

**Figure 3:**
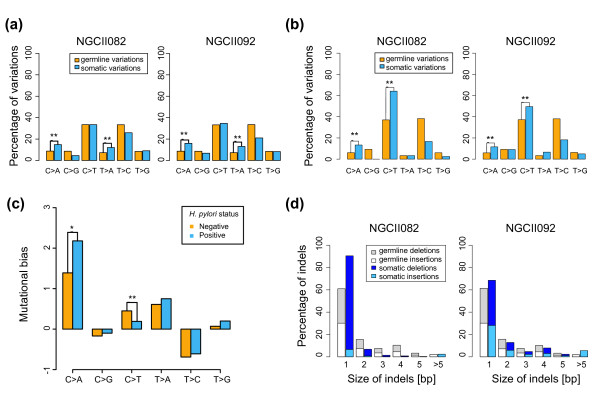
**Genome-wide and exome-wide mutational fingerprint**. **(a) **Frequency of various classes of somatic SNVs genome-wide. **(b) **Frequency of somatic SNVs exome-wide. **(c) **Mutational bias as a function of infection status using data from 34 exomes (bias for SNV class *i *was computed as (s_i _- g_i_)/g_i_, where s_i _and g_i _are the somatic and germline SNV frequencies). Note that nearly identical results were obtained when MSI tumors were excluded from the analysis (*P-value < 0.1; **P-values < 0.01, respectively). **(d) **Size-distribution of germline and somatic indels genome-wide.

We characterized mutational signatures in the GC genomes based on 14,856 somatic SNVs (11,738 indels) in NGCII082 and 17,473 somatic SNVs (2,486 indels) in NGCII092 that were identified from the whole-genome data (Table 1). This accounts for an average mutation frequency of 5 per megabase and included > 100 SNVs in protein coding regions for each tumor (Table 1; Note 6 in Additional file 1). Note that we identified more than five times the number of somatic variants uncovered in earlier sequencing studies [6,7] that were restricted to exomes (5,588 SNVs and 2,347 indels identified from 37 exomes), highlighting the statistical advantage of whole-genome analysis for studying mutational signatures. Overall, NGCII082, an MSI-positive tumor, displayed an excess of SNVs in protein coding regions (*P*-value < 0.02, χ^2 ^test) and a striking seven-fold higher frequency of micro-indels (Figures 2 and 3d) but a lack of large-scale SVs and amplifications or deletions (Figure 2 and Table 1). In contrast, NGCII092 exhibited a complex copy number profile of extensive focal amplifications and deletions, and a mutated *TP53 *gene, consistent with the presence of chromosomal instability (CIN) in the tumor genome (Figure 2). These results agree with the mutual exclusivity seen in MSI and CIN pathways for inducing mutations in other cancers as well [12].

The clear excess of micro-indels in the MSI-positive GC (Figure 3d; Figure S10 in Additional file 1) was characterized by a pattern of single base-pair thymine deletions in mononucleotide repeats (79%). In contrast, there were a comparable number of insertions in both the MSI-positive and CIN-positive GC, and a similar deletion-specific pattern has also been noted before [13]. Also, non-thymine and non-mononucleotide repeat deletions were not found to be in excess. The correlation between MSI phenotype and the specific deletion signature identified here was further confirmed from previous exome-sequencing data [7] (four MSI-positive exomes), though this aspect was not noted in the previous work. In terms of genomic location, the deletions were randomly scattered throughout the genome and occurred in proportion to the regional presence of thymine mononucleotide repeats (that is, 85% of homopolymers > 5 bp). Thus, despite the bias towards thymine deletions, there seems to be an absence of a targeting mechanism on the genome for the MSI-associated signature.

Despite exhibiting very different somatic alteration patterns (MSI or CIN), the mutational frequencies of both GCs at the single nucleotide level were highly similar, being significantly biased towards C > A and T > A alterations compared to normal genomes (*P*-value < 10^-16^, χ^2 ^test; Figure 3a). These alterations likely represent mutations caused by reactive oxygen and nitrogen species (ROS and RNS), which are known to produce C > A and T > A mutations [14]. Also, a likely trigger is *H. pylori *infection, which has been shown to cause chronic inflammation and ROS/RNS production in gastric epithelial cells [14]. The C > A mutations observed were associated with highly significant sequence-selectivity, being marked by an excess at CpCpT (NGCII082, odds ratio (OR) = 3.2, *P*-value < 10^-16^, χ^2 ^test) or TpCpA sites (NGCII092, OR = 1.7, *P*-value < 10^-16^, χ^2 ^test) and extensions of these motifs (Materials and methods; Note 6 and Figure S6 in Additional file 1 and Table S14 in Additional file 6). This pattern is distinct from the C > A signature seen in smoking-associated small-cell lung cancer where an excess was seen in CpG dinucleotides outside CpG islands, suggesting a link with methylation status [2,3]. Further work is required to identify the mechanistic basis of sequence selectivity in this genome-wide GC-specific signature.

#### Exome-biased mutational signature in GC

Unlike the MSI and ROS/RNS signatures that were present in coding and non-coding regions of the genome, we also detected a third GC mutational signature only evident in coding regions (Figure 3b), characterized by an excess of C > T mutations. These mutations were in excess at CpG (NGCII082, OR = 1.2, *P*-value < 10^-16^, χ^2 ^test) and GpC site (NGCII092, OR = 1.4, *P*-value < 10^-16^, χ^2 ^test) dinucleotides. The CpG alterations likely represent deamination of methylated cytosines followed by errors associated with transcription-coupled repair, which has also been observed in other cancers [2,4]. However, the latter bias towards C > T alterations occurring at GpC motifs appears to be a unique feature not previously reported in other cancers [2,4] and could represent deamination due to enzymes such as AID (activation-induced cytidine deaminase) [15]. AID is known to preferentially target transcribed regions [16] and is aberrantly activated due to *H. pylori *infection in the gastric epithelium [17]. Taken collectively, our whole-genome sequencing data implicates a minimum of three mutational signatures present in GC genomes, related to the presence of MSI, ROS/RNS, and deamination processes.

To further characterize the mutational signatures, we re-analyzed a total of 40 GC exomes, combining data from earlier studies [6,7] with two new exomes in this study (Materials and methods; Table S8 and Figure S7 in Additional file 1). Specifically, a comparison of somatic and germline frequencies for the exomes showed that all but one patient had a significant excess of C > A (ROS/RNS-related) or C > T (deamination-related) alterations and 23 GCs (> 50%) had an excess of both mutations (Fisher's exact test *P*-value < 0.01), establishing these two mutational classes as the most significant single-nucleotide alterations in GC. These patterns were independent of histological subtype (intestinal, diffuse and mixed-type) and MSI status (the excess is also seen in all but one non-MSI tumor). Moreover, the frequencies of C > T and C > A mutations were significantly different in GCs with active *H. pylori *infection compared to those lacking active infection (Wilcoxon rank sum test *P*-value < 0.006 and 0.06, respectively; Figure 3c). Overall, these results support the widespread role of ROS/RNS-associated C > A and deamination-associated C > T mutations in gastric cancer and are suggestive of their link to *H. pylori *infection.

A strong signature for transcriptional-coupled repair has been described before in other cancers [2,4] and our analysis also confirmed this in GC, in that poorly transcribed regions of the genome were associated with significantly more mutations (Figure S8 and Note 8 in Additional file 1). However, in contrast with earlier reports, we did not see a significant bias for mutations in the transcribed versus non-transcribed strand in most mutational classes (except for T > G, *P*-value < 0.05, χ^2 ^test; Figure S8 in Additional file 1). The absence of this latter pattern may be a consequence of the higher mutational burden from mutagens that also act in a transcription-coupled fashion (for example, AID [16]).

### Impact of mutational signatures on genes in GC

The overall impact of the mutational signatures identified here on gastric tumorigenesis is a complex question influenced by several factors, including the nature of mutations, the function of genes that are frequently impacted as well as genetic background and selection processes. We aimed to provide an initial assessment using two approaches: (i) by characterizing the proportion of genes affected by various mutational classes; and (ii) by identifying recurrently mutated genes in subtypes of GC defined by mutational processes.

Overall, a majority of mutated genes in NGCII082 were due to SNVs (77%) while CNVs and SVs played a dominant role in NGCII092 (82%) (Table 1). In total, we identified 107 SVs that affected genes by truncation, fusion, deletion, tandem duplication or rearrangements within the gene body. Ninety-six (90%) of these were identified in the CIN phenotype exhibiting tumor NGCII092, illustrating the genic burden from this mutational process. In contrast, small insertions and deletions (indels) were seen in few genes, even in the tumor with MSI phenotype (despite indels being roughly as common as SNVs genome-wide; Table 1), though their ability to cause frameshifts is likely to impact gene function more often than SNVs. Among SNVs, even though the deamination-related C > T signature is only seen in a small fraction of the genome, it plays a larger role in GC due to its targeted impact on genes. More than 48% of the non-synonymous mutations seen (48% in NGCII092 and 59% in NGCII082) in the two tumors were due to C > T mutations, compared to less than 19% for C > A mutations (Table 1). Among recurrently mutated genes in GC (Table S7 in Additional file 1 and Table S9 in Additional file 3), non-synonymous mutations in the tumor suppressor genes *TP53 *(mutated in 50% of samples) and *PTEN *(18% of samples), and oncogenes *PIK3CA *(13%; 8% have *PTEN *and *PIK3CA *mutations) and *CTNNB1 *(10%) were often in the form of C > T mutations (29%). This was also seen in several novel recurrently mutated genes such as *AQP7*, *SPTA1 *and *RP1L1 *(mutated in > 10% of tumors; Table S7 in Additional file 1).

Pathway analysis of mutated genes revealed that the two most enriched sets were β1-integrin mediated cell-surface interactions and signaling events mediated by class III histone deacetylases, a refinement of previous analysis [7] (Table S10 in Additional file 4). Furthermore, we identified genes implicated in *RAC1 *regulation to be mutated in 83% of *H. pylori *positive samples (*P*-value < 0.05 Fisher's exact test). *RAC1 *is a member of the Rho GTPase family known to play diverse oncogenic roles [18], shown to regulate the *H. pylori *virulence factor *VacA*, and known to promote vacuole formation in epithelial cells [19]. Mutations in the *RAC1 *pathway could thus simultaneously promote *H. pylori *infection as well as gastric tumorigenesis.

Finally, to further characterize the impact of mutational processes on genes in GC, we considered two specific subtypes for identifying recurrently mutated genes, MSI-positive GC and *TP53*-wild-type GC (Tables S11 and S13 in Additional file 1 and Table S12 in Additional file 5). We used *TP53*-wild-type status as a surrogate marker for tumors without the CI phenotype as *TP53 *is known to suppress chromosomal instability [20]. In this class of GCs, in addition to the tumor suppressor gene *PTEN *and *TTK *that interact with *TP53*, we identified *PAPPA*, a marker for pregnancies with aneuploid fetuses [21], as being recurrently mutated (Table S13 in Additional file 1; note that the average mutation rate for the whole-genome sequencing (WGS) samples in an approximately 2 Mbp window surrounding *PAPPA *is similar to the genome-wide rate, that is 5.3 versus 5.2 mutations/Mbp). A screen of an additional 94 gastric cancer/normal pairs confirmed the frequency of *PAPPA *mutations as being 6% among all GC samples (Table S12 in Additional file 5) and 20% among *TP53 *wild-type GCs (with mutations in key functional domains; Figures S13 and S14 in Additional file 1), highlighting it as a potential driver gene in this subtype.

In MSI-positive GCs, *ACVR2A*, *RPL22*, *LMAN1*, and *STAU2 *were observed to have recurrent single base thymine deletions in poly(T) regions (Table S11 in Additional file 1) and this was confirmed in a screen of an additional 94 gastric cancer/normal paired samples (9 MSI-positive; Table S12 in Additional file 5 and Figure S9 and Note 9 in Additional file 1). In total, *ACVR2A *was mutated in a region of 8 thymines in 86% of MSI-positive GCs tumors, *RPL22 *in a region of 8 thymines in 64%, *LMAN1 *in a region of 9 thymines in 50% and *STAU2 *in a region of 8 thymines in 29%. Based on the average frequency of mutations in homopolymer regions in the MSI-positive tumors (4.5% of 8 thymine stretches (*n *= 778) and 4.8% of 9 thymine stretches (*n *= 183), respectively, in exomic regions), mutations in *ACVR2A*, *RPL22 *and *LMAN1 *were in significant excess (Bonferroni-corrected *P*-value ≤ 0.0003, exact binomial test). In each gene, all the deletions occurred in the same homopolymer tract containing thymines, a pattern linked to the MSI phenotype, and none of the MSI-negative GC tumors carried these mutations. In contrast, mutations in the recently reported MSI-associated putative driver gene *ARID1A *were not restricted to deletions or MSI-positive tumors [7]. Interestingly, *ACVR2A *(encoding a TGF-β super-family differentiation factor) has been described to be recurrently mutated in MSI-positive colorectal cancer [22]. Also, the frequency of mutations seen here is comparable to the previously reported frequency in MSI-positive colorectal cancer [23,24] and emphasizes the importance of *ACVR2A *and TGF-β signaling in MSI-positive GC, while unraveling the oncogenic roles of *RPL22 *and *LMAN1 *requires further investigation.

## Discussion

Until long read sequencing of several kilo-base pairs is routine, the combination of SR and long fragment mate-pair sequencing remains the most powerful approach to comprehensively capture micro- and macro-scale alterations in the cancer genome. The combination of SR and DNA-PET sequencing in this study thus provides the first comprehensive assessment of somatic alterations in GC. In particular, our results highlight the importance of whole-genome analysis for reconstructing the lineage of complex somatic structural variants and characterizing mutational process and their genomic impact in cancer. For example, while point mutations in the *KRAS *gene have been well characterized, our whole-genome analysis enabled the first detailed reconstruction of amplification in the *KRAS *locus (a common event in GC) and a concomitant deletion of a proposed tumor suppressor gene *RASSF8*.

The analysis of several exome-sequencing datasets in earlier studies [6,7] was able to provide only a limited view of mutational processes in GC. Whole-genome analysis was essential for providing sufficient detail and statistics to identify the features and relative impact of the various mutational processes (for example, MSI, ROS/RNS and CI). This is best exemplified by the identification of a uniquely localized, deamination-linked mutational fingerprint whose significance would have been missed in an exome-based study. We further characterized the impact of this mutational process and identified the recurrently mutated genes *PAPPA*, *ACVR2A*, *RPL22*, *LMAN1*, and *STAU2 *in subtypes of GC defined by mutational processes.

## Conclusions

While computational tools for *de novo *cancer genome assembly are limited, its utility is demonstrated by our reconstruction of the *H. pylori *strain genome and assembly-based characterization of SVs and fusion genes at the base pair level. As sequencing costs continue to drop, whole-genome sequencing and assembly of affected tissues can serve as a tool for biomarker and pathogen discovery in cancer and other diseases. Assembly tools need to be refined to address the twin challenges of genomic amplifications and mixed cell populations and the availability of whole-genome SR and DNA-PET data from the clinical samples in this study should serve as a useful resource in this effort.

## Materials and methods

### Patient samples and clinical information

Patient samples and clinical information on tissue and blood samples were obtained from patients who had undergone surgery for gastric cancer at the National University Hospital, Singapore, and Tan Tock Seng Hospital, Singapore. Informed consent was obtained from all subjects and the study was approved by the Institutional Review Board of the National University of Singapore (reference code 05-145) as well as the National Healthcare Group Domain Specific Review Board (reference code 2005/00440). Clinical information for the two patients whose samples were analyzed by whole-genome sequencing is provided in Table S1 in Additional file 1 and additional information for the 94 gastric tumors used for targeted screening is provided in Table S12 in Additional file 5.

### Library preparation and sequencing

For WGS sequencing, genomic DNA isolated from tumor and blood samples was randomly fractionated using a Roche Nebulizer following the manufacturer's instructions (Madison, Wisconsin, USA). Fractionated DNA was then end-repaired, A-tailed at the 3' end, ligated with Illumina paired end adaptors, PCR amplified followed by gel-selection of a range of 400 to 600 bp fragments as templates and sequenced by Illumina GA from both ends to obtain 76 or 101 bp reads at each end (Table S2 in Additional file 1). DNA-PET libraries were constructed as described elsewhere [9] and were sequenced by the Applied Biosystems SOLiD system (Carlsbad, California, USA, Table S3 in Additional file 1). Exome sequencing was performed as described earlier using SureSelect Human All Exon Kit v1 (Agilent Technologies, Santa Clara, California, USA) and sequencing on two lanes of Illumina GA-IIx sequencer using 76 bp paired-end reads [6].

### Mapping and variant calling

Paired-end Illumina reads were mapped to the reference human genome (UCSC hg18) using ELAND (Illumina Inc.) and reads that failed pass-filter were removed from further analysis. SNVs and indels were called for each sample separately using SAMtools [25] (v0.1.7-6, SNP-quality threshold = 20, consensus-quality threshold = 30) (Table S4 in Additional file 1). Identical variant calls in tumor and matched normal samples were used to identify germline variants. Variant calls unique to the tumor, where the normal genotype called by SAMtools was different and where less than two reads of the variant genotype were seen in the normal sample, provided the list of somatic variants. Illumina reads from exome sequencing were analyzed using this pipeline after BWA [26] mapping (Table S8 in Additional file 1). As a control, we noted that germline SNV frequencies were nearly identical across all exomes from WGS and exome sequencing datasets (Figure S7 in Additional file 1). Somatic SNV frequencies and neighborhoods were compared to germline frequencies to assess enrichment. A neighborhood of up to 2 bp surrounding an SNV was used to identify enriched motifs. Somatic indel calls were required to be supported by at least 20% of the reads, by reads on both strands, with a minimum of 10 reads overlapping the position in the tumor and no indel calls in the normal sample. Somatic SNVs and indels in protein-coding regions and introns were confirmed by Sanger sequencing to have a high validation rate (83 SNVs, validation rate = 90%; 72 indels, validation rate = 96%). SNV neighborhood analysis was done by extracting 5 bp sequences upstream and downstream of mutations. Germline and somatic copy number variants were identified using the program RDXplorer [27] with default parameters.

DNA-PET tags were mapped individually to the reference human genome (UCSC hg18) in color space allowing two color code mismatches per tag by the SOLiD System Analysis Pipeline Tool Corona Lite (Applied Biosystems Inc.). Contigs of the reference sequence with unresolved location (random_chr) and alternative MHC haplotypes were excluded from the reference for mapping. Individually mapped tags were paired by Corona Lite. In cases where one or both tags had multiple mapping locations, a process termed 'rescuing' favored the creation of concordant PETs (both tags are on the same chromosome, same strand, same orientation, correct 5' → 3' order and in the expected distance to each other).

SVs, based on clusters of non-concordant PETs, were called using the GIS DNA-PET pipeline [9] with refined quality control criteria: (i) PET clusters of size < 6 were excluded; (ii) the regions to which the 5' and 3' tags of a cluster mapped had to be at least 1 kbp in size each; (iii) PET clusters that had a supercluster (connected component of overlapping clusters [9]) size > 100 required a higher cluster size of 10; and (iv) PET clusters with high sequence similarity between the two fused regions (BLAST score > 2,000 for 20 kbp windows around the predicted break points) were excluded. To distinguish between germline and somatic SVs, paired normal and tumor samples were compared as described previously [9]. Further filtering of known germline SVs and PCR validation are described in Note 1 in Additional file 1.

### Cancer genome assembly

Contig assembly, scaffolding and gap-filling of the Illumina sequencing data were done using the assembler SOAPdenovo [28]. DNA-PET reads were mapped to the SOAPdenovo assembly with Bowtie [29] and the resulting linking information was used to produce larger scaffolds based on the optimal scaffolder Opera [30]. Scaffolds and contigs were refined further with the gap-filling module in SOAPdenovo, employed for bridging scaffold gaps, where feasible. Using the SR reads alone, we obtained 12 kb scaffold N50 for both tumors. The DNA-PET reads allowed for improvement of assembly connectivity to a N50 of 65 kb and 41 kb for NGCII082 and NGCII092, respectively. Assemblies were compared to the reference human genome (UCSC hg18) using the MUMmer package [31] and alignments longer than 1 kbp were used to identify deletions and insertions larger than 20 bp. Overall, 12,861 deletions and 143 insertions were found in NGCII082 and 9,274 deletions and 108 insertions in NGCII092 of which 3 events > 2 kbp missed by DNA-PET analysis were identified in each sample. Fusion genes were validated and breakpoints were confirmed by using the gap-filling module in SOAPdenovo to bridge scaffolds constructed around the breakpoint. Sequences missing in the reference human genome were identified based on the criteria that they should be > 500 bp long and have no match to the reference genome with > 90% identity. Reads were mapped to the novel sequences using Bowtie to identify regions with no read coverage in the middle of a scaffold that could indicate a potential mis-assembly.

### Analysis of microbial sequences

Reads with a putative microbial or viral origin were identified by mapping reads with no mapping to the human genome, to a database of complete bacterial and viral genomes in NCBI (using Bowtie [29]). Matches were filtered for low-complexity sequences (more than three matches of any 5-mer) and the remaining reads were used to estimate the abundance for each species (pooling reads mapped to different strains of a species). Each species was checked for multiple distinct read matches to its genome (> 4 distinct regions, where the genome was segmented in 1 kbp windows) and the presence of unique read matches (using the unique option in Bowtie). The small fraction of reads of putative bacterial origin in the matched blood samples (possibly reagent contamination) were used as control and read matches to the corresponding species were excluded in determining the tumor associated microbiome. Concentration of *H. pylori *cells in relation to tumor cells was estimated based on the assumption of uniform coverage of both cell types, where coverage = k × Number of cells × Size of genome, for a constant k and the populations are assumed to be clonal.

### Functional annotation of SNVs and indels

For all samples, SNV and indel calls were annotated using the SeattleSeq server [32] and SIFT [33], respectively. Pathway analyses were performed based on non-synonymous SNVs and indels using the Pathway Interaction Database [34] (sample pfg005T from Wang *et al. *[7] was excluded as it only had four somatic mutations).

### Data access

Sequencing data for this publication have been deposited in NCBI's Gene Expression Omnibus [35] and is accessible through GEO Series accession number GSE30833.

## Abbreviations

CIN: chromosomal instability; CNV: copy-number variation; DNA-PET: DNA paired-end tag; GC: gastric cancer; MSI: microsatellite instability; OR: odds ratio; RNS: reactive nitrogen species; ROS: reactive oxygen species; SNV: single nucleotide variation; SR: short read; SV: structural variation; WGS: whole-genome sequencing.

## Authors' contributions

YR and KGY initiated the study. NN, DB, AMH, PBOT and YR designed the experiments. JR, MT, FZ, JBYS, RS and KGY obtained ethical approval, patient information and patient samples and commented on clinical relevance of genomic findings. ASMT, ZZ and AH constructed genome-wide sequencing libraries (SR and DNA-PET). NN, DB and AMH coordinated the data analysis. DB and NN did the mutation analysis with assistance from LV and AS. PEJ did the expression analysis. AMH, FY, WHL, PNA, XYW and CCK did the copy number and structural variation analysis with guidance from WKS, GB and MLH. FY, ASMT and YYS performed validation of structural variations and point mutations and screened for recurrent mutations and indels. YYS performed quantitative PCR. SG and DB did the assembly analysis with guidance from NN. DB analyzed the impact of mutations with guidance from AMH, NN, PBOT and KVD. MW, SYC, BP and RS performed microsatellite instability analysis for the cohort of patient samples that were screened for recurrent mutations. XR coordinated Illumina and SOLiD sequencing of the WGS samples. ZJZ, IC, CKO, ND, BTT, SR and PBOT coordinated and executed the exome sequencing and mapping analysis of the data. NN, DB, AMH and PBOT wrote the manuscript. All authors read and approved the final manuscript.

## Supplementary Material

Additional file 1 **Supplementary Methods, Tables and Figures**. Click here for file

Additional file 2 **Table S6. Details of somatic SVs identified by DNA-PET in gastric tumors NGCII082 and NGCII092**. Click here for file

Additional file 6 **Table S14. Enriched bases and motifs in the neighbourhood of C > A mutations**. Click here for file

Additional file 3 **Table S9. Genes recurrently mutated by non-synonymous SNVs or indels in four or more patients out of 40 GC exomes**. Click here for file

Additional file 4 **Table S10. Enriched functions and pathways in Gastric Cancer**. Click here for file

Additional file 5 **Table S12. Screen for recurrent mutations in 94 GC tumor/normal pairs by Sanger sequencing**. Click here for file
