# Supplementary material for: Whole-genome reconstruction and mutational signatures in gastric cancer
Source: Genome Biol. 2012 Dec 13;13(12):R115. doi: 10.1186/gb-2012-13-12-r115 (PMC4056366; doi:10.1186/gb-2012-13-12-r115)
Supplement: Additional file 1 — Supplementary Methods, Tables and Figures. [file gb-2012-13-12-r115-S1.DOCX]

Whole-genome reconstruction and mutational signatures in gastric cancer

Niranjan Nagarajan, Denis Bertrand, Axel M Hillmer, Zhi Jiang Zang, Fei Yao, Pierre- Étienne Jacques, Audrey SM Teo, Ioana Cutcutache, Zhenshui Zhang, Wah Heng Lee, Yee Yen Sia, Song Gao, Pramila N Ariyaratne, Andrea Ho, Xing Yi Woo, Lavanya Veeravali, Choon Kiat Ong, Niantao Deng, Kartiki V Desai, Chiea Chuen Khor, Martin L Hibberd, Atif Shahab, Jaideepraj Rao, Mengchu Wu, Ming Teh, Feng Zhu, Sze Yung Chin, Brendan Pang, Jimmy BY So, Guillaume Bourque, Richie Soong, Wing-Kin Sung_,_ Bin Tean Teh, Steven Rozen, Xiaoan Ruan, Khay Guan Yeoh, Patrick BO Tan, Yijun Ruan

**Note 1: Filtering of SVs and PCR validation**

To further filter germline SVs in the tumors which were missed in the paired normal sample, we used SVs identified by paired-end sequencing in an additional 29 unrelated normal individuals (20 individuals analysed by DNA-PET and 9 individuals analysed by other paired-end sequencing protocols [1, 2]). Somatic SV calls were validated by PCR and Sanger sequencing (100 SVs, validation rate = 81%). Note that the estimated validation rate is likely to be a lower bound on the true rate as in 14% of the cases, failure to obtain PCR product for tumor and blood samples was interpreted as a false positive, but could also be due to other reasons for PCR failure. The program breakdancer [[3]](#_ENREF_3) was also used to call SVs using the WGS datasets (default parameters). Only 57% of PCR validated SVs (and 3 out of 6 validated fusion genes: *OVCH1*-*CCDC91*, *COPG2*-*AGBL3*, *ZC3H15-ITGAV*) were identified by this analysis (based on overlap with a 500 bp window surrounding the breakpoint), highlighting the utility of DNA-PET libraries in calling SVs in repeat rich regions. Note that, despite their differences, both tumors showed somatic rearrangements in two genes, *FHIT* and *WWOX* (three intragenic deletions and four complex rearrangements in *FHIT* and four deletions in *WWOX*, **Additional File 2, Table S6**) confirming the fragility of these loci in gastric cancer.

Note 2: Cancer Genome Assembly

For the tumors sequenced in this project, the availability of a large-insert (~10 kbp) library with high physical coverage (>130X), in addition to nearly 30X base-pair coverage from WGS reads of short-insert libraries provides a unique test-bed for *de novo* tumor genome reconstruction. As a proof-of-principle, we constructed highly-contiguous draft assemblies for tumor as well as normal genomes (**Table S5**). Alignment of the assembly to the reference genome aided in identifying SVs missed by DNA-PET analysis (e.g. a 100 kbp germline deletion of the gene *BC073807* in both patients), delineating breakpoint sequences for fusion genes and reconstructing regions missing in the reference human genome (3 Mbp in total for each tumor). Novel sequences found in the tumor genomes were also found in the normal genomes (and vice versa) with the sole exception being the contaminant phage genome (phi-X174, used as control in Illumina sequencing) in NGCII082. Also, an additional set of 21 (33) somatic SNVs and 1727 (1298) germline SNVs were called for NGCII082 (NGCII092) in the novel sequences and potentially genic regions in the novel sequences were annotated using BLAST matches (**Table S5**). Potential mis-assemblies were also identified (and excluded from the reported results) in these sequences by a mapping based analysis to identify regions with no fragment coverage. We envisage that, with further refinement in assembly techniques, this *de novo* ability to reconstruct and study tumor and cell-line genomes will be invaluable for transcriptional regulation and systems-biology studies of cancer genomes.

**Note 3: Reconstruction of genomic rearrangements**

The combined SR/DNA-PET data (Methods) enabled a detailed putative reconstruction of the evolutionary lineage of the amplified *KRAS* locus. Specifically, for chromosome 12p we observed i) a somatic 1.9 Mbp deletion centromeric to *KRAS* as an early event in the lineage of cells subsequently acquiring *KRAS* amplification (Figure 1b), ii) an accumulation of unpaired inversions with a short distance between their breakpoints consistent with breakage-fusion-bridge (BFB) cycle based amplification [[4]](#_ENREF_7) (Figure S1), and iii) a concomitant deletion of *RASSF8*, a proposed tumor suppressor gene, within the same amplicon. The architecture of this 12p amplicon suggests that multiple rounds of BFB in this genomic locus may have resulted in both *KRAS* amplification and selective exclusion of *RASSF8* from the amplification process, ultimately enhancing the oncogenic potential of the resulting cellular lineage. RASSF8 has been shown to play a role in growth suppression through regulation of cell-cell contact in lung cancer cell lines [5]. In an independent dataset [[6]](#_ENREF_8), we observed multiple tumors exhibiting discernible copy number transitions between *KRAS* and *RASSF8*, supporting the assumption of an oncogenic effect of this structural feature.

The tumor of patient NGCII092 displayed an amplicon on 6p which was also marked by a sharp increase in copy number at the telomeric side and a gradual decline towards the centromere, as expected from amplification by BFB cycles (**Figure S2**). The corresponding core amplified region contains fourteen genes (estimated copy number >10), including the over-expressed gene *PAK1IP1* (data not shown), a negative regulator of the PAK1 kinase with a known role in interfering with NFKB signalling pathways [[7]](#_ENREF_4) and hence a plausible candidate for the driver in the amplification. Note that both amplicon regions, chromosome 6p and 12p, contain several types of intra-chromosomal rearrangements (in addition to the unpaired inversions) indicating that other mechanisms may have further contributed to the amplification and rearrangement of these loci (**Figures 1**, **S1** and **S2**).

**Note 4: Prediction of fusion genes**

Selective advantages provided by fusion oncogenes is a cancer driving mechanism and rearrangements constructing six fusion genes (*ZC3H15-ITGAV, COPG2-AGBL3, INTS4-RSF1, OVCH1-CCDC91, SOX5-OVCH1* and *YWHAB-BCAS1*) were observed in NGCII092 (and none in NGCII082). All rearrangements underlying fusion genes were validated by genomic PCR and Sanger sequencing and two fusion genes, *INTS4-RSF1* and *COPG2-AGBL3*, were found to be expressed by RT-PCR and Sanger sequencing (**Figure S3**). Two of the fusion genes were found in the *KRAS* amplification locus (one of these, *SOX5-OVCH1*, was missed in the WGS data), highlighting the role of amplicons as “foundries” for forging fusion genes. The gene, *OVCH1* encoding a secreted protease was involved in both fusions, *SOX5-OVCH1* and *OVCH1-CCDC91* (**Figure 1c**).

One of the fusion events observed is the product of a complex rearrangement on chromosome 20 connecting the genes *BCAS1* and *DOK5* over a distance of 720 kbp, but with no apparent focal amplification (**Figure S4**). The new fusion contains five DNA fragments that are 0.2 kbp to 8.6 kbp in size. Two of these fragments originate from intronic regions of the gene *YWHAB* that is highly expressed in 13 investigated gastric tumors and is located 9 Mbp upstream of *BCAS1*. The long span of DNA-PET data indicated that the five rearrangement points are located on the same DNA molecule (confirmed by targeted PCR/Sanger sequencing). The structure of this complex rearrangement resembles the pattern created by replication coupled mechanisms [[8]](#_ENREF_6). These mechanisms have been described for congenital disorders but cancer rearrangement points have recently been correlated with replication time points suggesting that these mechanisms contribute to somatic rearrangements in cancer[[9]](#_ENREF_7). Chromothripsis, a recently described cancer rearrangement mechanism in which a single catastrophic event creates new joins of many genomic fragments at one single time point, seems less likely to be the underlying mechanism here since these rearrangements resulted in multiple copies of the same fragment (**Figure S4**).

Note 5: Identification of Sequences of Microbial Origin

The unbiased nature of shotgun sequencing data obtained from patient tumor samples provides a unique resource to study not only the tumor genome but also associated microbial genomes. While previous cancer genome studies have not reported the finding of microbial sequences [[10-14]](#_ENREF_8), gastric cancer provides a unique setting due to its well-known association with *H. pylori*. In fact, our sequencing data does confirm the presence of an active infection in the sample NGCII082 with 2114 WGS reads (out of 1.0 billion) and 662 DNA-PET tags identified to be of *H. pylori* origin, with an estimated concentration of 1 per 100 tumor cells (see **Methods)**. Strikingly, no reads for NGCII092 (out of 1.2 billion) were found to be of *H. pylori* origin, confirming the histological report for the tumor at a molecular level. These results also provide a proof-of-concept for the adoption of whole-genome sequencing as a routine tool to aid pathogen discovery in cancer and other diseases.

When combined, the WGS reads and DNA-PET data provide ~1X physical coverage and 0.2X base-pair coverage of the *H. pylori* genome. While this information is insufficient for *de novo* reconstruction of the infecting strain, the gross structure of the genome as well as the presence of genes and pathogenicity islands can still be inferred (**Figure S5**). In particular, the sequence data confirms the presence of the *cag* island (an important pathogenicity locus encoding type IV secretion system proteins [15, 16]) and individual genes such *cagE* and *cagG* whose role in inducing pro-inflammatory cytokines in gastric epithelial cells has been described before [17, 18]. The presence of the *cag* island as well as the genes *vacA* (a vacuolating cytotoxin) and *babA* (encodes an antigen-binding outer membrane protein), all three of which are important risk factors for gastric cancer [19, 20], further highlights the virulence potential of the *H. pylori* strain infecting the tumor.

While colonization by *H. pylori* typically establishes a monoculture in the stomach [[21]](#_ENREF_19), persistent colonization is known to decrease acid secretion [[22]](#_ENREF_20) and the altered stomach environment can facilitate the proliferation of other species [[23]](#_ENREF_21). Strikingly, in the case of the *H. pylori* infected tumor (NGCII082), the sequencing data confirms the presence of *H. acinonychis*, *E. coli* and several *Lactobacilli* (**Figure S5**) and, in contrast, this flora is not found in the *H. pylori*-deficient tumor (NGCII092). The presence of *Lactobacilli* is intriguing as several species have been shown in *in vitro* studies to have the potential to suppress the growth of *H. pylori* [24, 25]. To our knowledge, this is the first example of a bacterial pathogen genome and a tumor-associated microbiome being characterized directly from tumor sequencing data.

Note 6: Frequencies of Somatic Mutations

For each patient, tumor and normal genomes were compared to the reference human genome (UCSC hg18) and to each other to obtain a list of tumor-specific somatic variants as well as germline variants (**Methods**). For somatic SNVs, 14,856 variants were inferred for NGCII082 and 17,473 for NGCII092 with an average mutation frequency of 5 per megabase (**Table 1**). This mutation frequency is significantly greater than that observed in prostate cancer [[10]](#_ENREF_8) (0.9 per megabase), similar to the rates in breast cancer, acute myeloid leukemia and hepatocellular carcinoma [11, 13, 14, 26], and lower than observed frequencies in lung cancer and melanoma [12, 27] (10-30 per megabase). While NGCII082 had fewer somatic SNVs across the entire genome, a comparison of mutations in protein coding regions revealed a higher proportion of somatic variants in comparison to NGCII092 (p-value < 0.02, χ^2^ test). The proportion of non-synonymous to synonymous variants was also higher in NGCII082 (2.66:1 vs 1.7:1), but comparable to that found in previous studies [27, 28] and not significantly different from that expected by chance (p-value > 0.3), suggesting that a majority of variants do not provide a selective advantage. For indels, the MSI-positive NGCII082 had slightly fewer insertions (943 vs 1,090) but more than 7 times the number of micro-deletions genome-wide (10,795 vs 1,397). In contrast, when analyzed at the level of large somatic SVs and CNVs (> 1 kbp), NGCII092 was revealed to be much more aberrant than NGCII082 (**Table 1**, 146 vs 12 SVs and 21,776 vs 836 CNVs). These results demonstrate that individual gastric cancers, despite being histologically similar, can nevertheless exhibit strikingly distinct mutational profiles and is in accordance with previous observations that chromosome and microsatellite instability are mutually exclusive mutation patterns [[29]](#_ENREF_28) (**Figure 2**).

The genomic neighbourhood of somatic mutations in the two WGS tumors reflect characteristic patterns in C>A and C>T mutations and commonalities in all other classes. Detailed analysis of the neighbourhood around C>A mutations suggests that in addition to the enrichment of certain bases in the neighbourhood of the mutation, certain combinations of bases are also enriched (**Additional File 6, Table S14**). These motifs might represent the structural features recognized by a potential mutagen.

The shared genomic neighbourhoods between the WGS tumors included an excess of T>G mutations at YpTpT sites (OR = 1.9, p-value < 10^-16^, exact binomial test), T>A mutations enriched in AT-rich regions (WpTpW sites, OR > 1.3, p-value < 10^-16^, exact binomial test), C>G mutations in AT-rich regions (WpCpW sites, OR > 1.2, p-value < 10^-16^, exact binomial test) and T>C mutations at TpTpT and ApTpA tri-nucleotides (OR = 1.4, p-value < 10^-16^, exact binomial test). As control, we noted that the genomic neighbourhood of germline SNVs was nearly identical and similar to what has been reported previously [[12]](#_ENREF_3).

Note 7: Variant Annotation

At the genic level, an overlap of known susceptibility variants for gastric cancer from the Human Gene Mutation Database [[30]](#_ENREF_29) with germline variants seen in the two WGS patients revealed that nearly all previously-identified risk variants were shared. In particular, both patients share homozygous deletions of *GSTM1* that have been linked with increased cancer susceptibility [[31]](#_ENREF_30), variants in *XRCC1* (R280H and G399R) that have been implicated in impaired function [32, 33] of this important base excision repair gene and alleles (R1826H and D2937Y) in *VCAN* that have been associated with reduced susceptibility to intestinal-type gastric cancer [[34]](#_ENREF_33). Interestingly, the patients also share an *ERBB2* variant (I655V) associated with several breast cancer phenotypes [[35]](#_ENREF_34). Only two germline susceptibility variants were found to be unique (to NGCII092) – a third variant in *XRCC1* (R194W) and a variant in the mismatch repair gene *MSH6* (E1163V) linked to hereditary colorectal cancer [[36]](#_ENREF_35).

Among somatic SNVs, as expected, there is little overlap between the two WGS tumors, though at the genic level, both tumors have a non-synonymous SNV in the Nebulin gene (P5179L in NGCII082 and T6162M in NGCII092). While mutations in Nebulin have previously not been reported to have a role in gastric cancer, interestingly in a recent proteomic study, Nebulin was found to be one of ten over-expressed proteins in gastric cancer [[37]](#_ENREF_36). Other non-synonymous SNVs in the two tumors were predicted to be function-altering for a wide-spectrum of known oncogenes and tumor suppressors with a role in gastric cancer. NGCII082, in particular, has SNVs affecting several classic oncogenes including *PIK3CA*, *CTNNB1* and *ROS1* (P1679Q) (with known associations to gastric cancer) as well as a frameshift-causing indel in the tumor suppressor *PTEN*. The *PTEN* frameshift mutation in NGCII082 is located in exon 7 and correlates with lower expression values for exons 7 to 9 (**Figure S11a**). In NGCII092, several tumor suppressor genes have SNVs affecting them including *TP53*, *PDGFRB*, *CASP10* (alterations of *CASP10* are commonly found in gastric cancer and may affect its apoptotic function [[38]](#_ENREF_37)) and *SMAD4* (S178*, loss of *SMAD4* expression has been correlated with progression of gastric cancer [[39]](#_ENREF_38)). The presence of S178* in NGCII092 also correlates with lower expression of *SMAD4* (**Figure S11b**). Nonsense mutations in the tumors include one affecting *ZC3H8* in NGCII092 (in addition to *SMAD4*) and mutations in *ABCA2* (associated with lipid transport and drug resistance in cancer cells [[40]](#_ENREF_39)), *CDC5L* (belongs to the spliceosome complex [[41]](#_ENREF_40)), *DIDO1* (associated with myeloid neoplasms [[42]](#_ENREF_41)) and *PTPN11* in NGCII082.

A small subset of the non-synonymous SNVs were also characterized *in silico* as being possible drivers of tumorigenesis affecting the genes *CTNNB1*, *CLK3* (a component of the splicing machinery), *TFE3* (frequent partner in oncogenic fusions in renal cell carcinoma [[43]](#_ENREF_42)) and *RANBP2* (a nucleoporin) in NGCII082 and *KALRN*, *PRMT3* and *GNAO1* (all three proteins have guanosine-binding function) in NGCII092. NGCII082 also has somatic alterations in two important DNA-repair genes, a non-synonymous SNV in *ERCC6*, an essential factor involved in transcription-coupled nucleotide excision repair [[44]](#_ENREF_43) (enabling RNA Pol II-blocking lesions to be rapidly removed from the transcribed strand of active genes) and a 2 bp deletion leading to a frameshift in *TOPBP1* which plays an important role in the rescue of stalled replication forks [[45]](#_ENREF_44).

For all samples, SNV and indel calls were annotated using the SeattleSeq server (<http://gvs.gs.washington.edu/SeattleSeqAnnotation/>) and SIFT [[46]](#_ENREF_46), respectively. Driver genes were predicted using the program CanPredict [[47]](#_ENREF_47) and filtered for mutations predicted to be tolerated by PolyPhen-2 [[48]](#_ENREF_48).

**Note 8: Expression Analysis**

Gene expression levels were determined on the Affymetrix U133 plus microarray according to the manufacturer’s recommendation. Raw expression data was jointly normalized for all samples (2 WGS samples and 11 additional tumors and a GC cell line) by the RMA algorithm [[49]](#_ENREF_49) on all probes using the BRB Array software. For *PTEN* and *SMAD4* transcript analysis, mapping location of individual probe sets was used to discriminate between transcripts. Differential expression of probe sets was called based on the criteria of 4 fold change of the normalized data (231 up-regulated and 123 down-regulated genes in the comparison between NGCII082 and NGCII092). Overall, expression levels for the tumors were remarkably well correlated (correlation = 0.97), but significant enrichment for differentially expressed genes was seen in a set of 20 genes (19 out of 20 up-regulated in NGCII082, p-value < 10^-16^, Fisher’s exact test) known to be up-regulated in advanced gastric cancer [[50]](#_ENREF_50) and consistent with the clinical information for NGCII082 (stage 3b with lymph node metastasis vs stage 1b and no lymph node metastasis for NGCII092). This correlation was also seen in a clustering analysis of NGCII082 and NGCII092 with 11 gastric tumors (based on the set of differentially expressed genes in the two tumors), where NGCII082 clustered with the patients known to have had tumors that metastasized (**Figure S12**).

**Note 9: Screening of 94 gastric cancer/normal pairs by Sanger sequencing**

To test for recurrence of single T deletions in poly(T) stretches of *ACVR2A*, *RPL22*, and *LMAN1*, DNAs of 94 gastric cancer tumors and paired normal gastric tissues were analyzed by PCR amplification of the genomic regions containing the poly(T) stretches followed by Sanger sequencing. Sequencing chromatograms were investigated manually. Frame shifts were observed in tumors only, suggesting that they were due to single base deletions in the template rather than due to sequencing/amplicon artifacts (**Figure S9**).

We screened the 94 tumor/normal pairs for mutations in *PAPPA* by PCR amplification of coding exons including exon/intron boundaries followed by Sanger sequencing and manual inspection of chromatograms. PCR assays could be established for all coding exons except exon 1 which was excluded from this analysis. The 94 tumor samples were assayed for MSI status by the MSI Analysis System (Promega) according to the manufacturer’s recommendations (**Additional File 5, Table S12**).

**The following supplementary tables can be found as separate Excel spreadsheets:**

**Table S6.** Details of somatic SVs identified by DNA-PET in gastric tumors NGCII082 and NGCII092 (**Additional File 2**).

**Table S9.** Genes recurrently mutated by non-synonymous SNVs or indels in four or more patients out of 40 GC exomes (**Additional File 3**).

**Table S10.** Enriched functions and pathways in Gastric Cancer (**Additional File 4**).

**Table S12.** Screen for recurrent mutations in 94 GC tumor/normal pairs by Sanger sequencing (**Additional File 5**).

**Table S14.** Enriched bases and motifs in the neighbourhood of C>A mutations (**Additional File 6**).

Table S1. Clinical information for GC patients with samples analyzed by whole genome sequencing.

| Patient ID | NGCII082 | NGCII092 |
| --- | --- | --- |
| Ethnicity and Gender | Chinese Male | Chinese Female |
| Age at surgery (years) | 77 | 77 |
| Tumor Stage (AJCC 6th Ed.) | 3b, No distant metastasis | 1b, No distant metastasis |
| Subtype and Grade | Intestinal, Tubular, Moderately Differentiated | Intestinal, Tubular, Moderately Differentiated |
| Other Features | Ex-smoker, *H. pylori* Infection, Chronic Gastritis and MSI^§^ | Chronic Gastritis and Intestinal Metaplasia^#^, Dysplasia |

^§^ 4 out of 5 homopolymer alterations (Promega) and loss of MLH1 and PMS2 expression based on tumoral immunohistochemistry.

^#^ *H. pylori* infections are highly prevalent in South East Asia [[51]](#_ENREF_51) and the presence of intestinal metaplasia [[21]](#_ENREF_19) suggests that the female patient is likely to have had a *H. pylori* infection in the past.

Table S2. Whole genome sequencing statistics.

| Patient ID | | NGCII082 | | NGCII092 |
| --- | --- | --- | --- | --- |
| Tumor | Bases Sequenced (in Gbp) | | 99 | 120 |
|  | Coverage | | 33 | 40 |
| Normal | Bases Sequenced (in Gbp) | | 145 | 139 |
|  | Coverage | | 48 | 46 |

Table S3. DNA-PET sequencing statistics.

| Patient ID | Library ID | Tissue | Tags | Mappable Tags | Tags (NR^1^) | PETs (NR^1^) | Span [bp] | Median span [bp] | cPETs^2^ | Coverage^3^ | dPETs^4^ | SVs^5^ |
| --- | --- | --- | --- | --- | --- | --- | --- | --- | --- | --- | --- | --- |
| NGCII082 | IHH045 | Blood | 457,934,506 | 232,476,585 | 157,940,191 | 60,202,263 | 8851-11656 | 10,274 | 54,065,950 | 195 | 6,136,313 | 182 |
|  | IHG021 | Tumor | 639,483,531 | 364,255,401 | 250,869,456 | 44,119,792 | 7590-11454 | 9,613 | 40,003,428 | 135 | 4,116,364 | 499 |
| NGCII092 | IHH046 | Blood | 515,066,446 | 262,070,237 | 173,353,894 | 66,989,856 | 8544-10959 | 9,889 | 61,016,144 | 212 | 5,973,712 | 612 |
|  | IHG028 | Tumor | 547,319,437 | 369,012,131 | 255,473,561 | 59,735,674 | 9372-13310 | 11,375 | 55,694,948 | 222 | 4,040,726 | 594 |

^1)^ non redundant
^2)^ concordant PET
^3)^ physical coverage
^4)^ non-concordant PET
^5)^ structural variations called based on quality curated PET clusters

Table S4. Variant calls for tumor and normal genomes for WGS data.

|  | Tissue | NGCII082 | NGCII092 |
| --- | --- | --- | --- |
| SNVs | Blood | 3,605,248 | 3,568,180 |
|  | Tumor | 3,509,704 | 3,603,777 |
| Indels | Blood | 390,744 | 380,300 |
|  | Tumor | 365,905 | 381,216 |
| CNVs | Blood | 145,878 | 146,129 |
|  | Tumor | 124,060 | 168,931 |

Table S5. Tumor & normal genome assembly statistics.

|  | NGCII082 | | NGCII092 | |
| --- | --- | --- | --- | --- |
|  | Tumor | Normal | Tumor | Normal |
| Assembled Length (in Gbp) | 2.6 | 2.7 | 2.6 | 2.6 |
| Contig N50^#^  (in kbp) | 18 | 28 | 17 | 18 |
| Largest Contig  (in Mbp) | 0.28 | 0.48 | 0.36 | 0.28 |
| Number of Contigs | 424,605 | 326,195 | 420,974 | 402,176 |
| Scaffold N50^#^  (in kbp) | 65 | 148 | 41 | 122 |
| Largest Scaffold  (in Mbp) | 1.02 | 1.42 | 1.02 | 1.3 |
| Number of Scaffolds | 302,975 | 222,068 | 329,350 | 232,361 |
| Protein Matches in Novel Sequences | BAB13908.1 (unnamed protein product),  BAD98065.1 (bitter taste receptor T2345), EAW98672.1(cytokine receptor-like factor 2) | | AAD14429.1 (prorelaxin), ADQ01558.1 (immunoglobulin heavy chain variable region), BAD98078.1 (bitter taste receptor T2R46), BAH12720.1 (unnamed protein product), CAA32540.1 (unnamed protein product), | |

^#^ Median length where more than half the assembled genome is composed of sequences of equal or greater length

**Table S7.** Recurrently mutated genes in Gastric Cancer. Genes are sorted by the number of samples (out of 40) with non-synonymous mutations normalized by the size of the coding region for the gene.

| **Gene ID** | **Gene Name** | **Length** | **# of mutated**  **samples** | **# of mutated samples/ Length** |
| --- | --- | --- | --- | --- |
| ***TP53*** | cellular tumor antigen p53 isoform b | 1182 | 20 | 0.01692 |
| ***PTEN*** | phosphatidylinositol-3,4,5-trisphosphate | 1212 | 7 | 0.00578 |
| ***AQP7*** | aquaporin-7 | 1029 | 4 | 0.00389 |
| ***ACVR2A*** | activin receptor type-2A precursor | 1542 | 4 | 0.00259 |
| ***STAU2*** | double-stranded RNA-binding protein Staufen | 1713 | 4 | 0.00234 |
| ***CTNNB1*** | catenin beta-1 | 2346 | 4 | 0.00171 |
| ***PIK3CA*** | phosphatidylinositol-4,5-bisphosphate 3-kinase | 3207 | 5 | 0.00156 |
| ***TTK*** | dual specificity protein kinase TTK isoform 1 | 2574 | 4 | 0.00155 |
| ***COPB2*** | coatomer subunit beta' | 2721 | 4 | 0.00147 |
| ***DHX36*** | probable ATP dependent RNA helicase DHX36 | 3027 | 4 | 0.00132 |
| ***CCDC73*** | coiled-coil domain-containing protein 73 | 3240 | 4 | 0.00123 |
| ***PCDH15*** | protocadherin-15 isoform CD3-2 precursor | 5889 | 6 | 0.00102 |
| ***FMN2*** | formin-2 | 5169 | 5 | 0.00097 |
| ***ARID1A*** | AT-rich interactive domain-containing protein 1A | 6858 | 6 | 0.00087 |
| ***PAPPA*** | pappalysin-1 preproprotein | 4884 | 4 | 0.00082 |
| ***SPTA1*** | spectrin alpha chain, erythrocyte | 7260 | 5 | 0.00069 |
| ***RP1L1*** | retinitis pigmentosa 1-like 1 protein | 7203 | 5 | 0.00069 |
| ***EVPL*** | envoplakin | 6102 | 4 | 0.00066 |

Table S8. Exome sequencing statistics for the samples in Zhang *et al*. [[52]](#_ENREF_52) compared to the additional samples in this study.

| Patient ID | Tumor  Type/  *H. pylori* status | Tissue | Bases  Sequenced (in Gbp) | Coverage | SNVs  Coding regions | SNVs  Non-synonymous |
| --- | --- | --- | --- | --- | --- | --- |
| 2000362 | Intestinal/  Negative | Blood | 8.7 | 126 | - | - |
|  |  | Tumor | 7.5 | 109 | 214 | 147 |
| 2000619* | Other/  Negative | Blood | 8.5 | 124 | - | - |
|  |  | Tumor | 8.7 | 127 | 184 | 128 |
| 2000778* | Other/  Positive | Blood | 8.5 | 124 | - | - |
|  |  | Tumor | 7.4 | 108 | 296 | 197 |
| 31231321 | Diffuse/  Positive | Blood | 9.8 | 143 | - | - |
|  |  | Tumor | 9.4 | 137 | 304 | 195 |
| 76629543 | Intestinal/  Positive | Blood | 8.5 | 124 | - | - |
|  |  | Tumor | 8.9 | 130 | 307 | 199 |
| 970010 | Intestinal/  Positive | Blood | 7.5 | 110 | - | - |
|  |  | Tumor | 7.9 | 115 | 218 | 140 |
| 980417 | Diffuse/  Positive | Blood | 10.0 | 146 | - | - |
|  |  | Tumor | 10.1 | 148 | 186 | 110 |
| 98748381 | Other/  Positive | Blood | 8.0 | 117 | - | - |
|  |  | Tumor | 7.9 | 115 | 171 | 121 |
| 990090 | Intestinal/  Negative | Blood | 8.5 | 124 | - | - |
|  |  | Tumor | 8.5 | 124 | 98 | 57 |
| 990098 | Intestinal/  Positive | Blood | 6.0 | 87 | - | - |
|  |  | Tumor | 7.2 | 104 | 205 | 133 |
| 990172 | Intestinal/  Positive | Blood | 8.6 | 124 | - | - |
|  |  | Tumor | 8.7 | 127 | 123 | 76 |
| 990300 | Intestinal/  Negative | Blood | 9.4 | 137 | - | - |
|  |  | Tumor | 8.6 | 125 | 270 | 143 |
| 990355* | Diffuse/  Positive | Blood | 8.9 | 130 | - | - |
|  |  | Tumor | 9.1 | 133 | 78 | 49 |
| 990396 | Diffuse/  Negative | Blood | 10.9 | 159 | - | - |
|  |  | Tumor | 9.8 | 143 | 295 | 184 |
| 990475 | Intestinal/  Positive | Blood | 8.9 | 130 | - | - |
|  |  | Tumor | 8.7 | 127 | 147 | 92 |
| 990515 | Intestinal/  Positive | Blood | 9.0 | 132 | - | - |
|  |  | Tumor | 6.3 | 91 | 281 | 203 |

^*^ Additional samples not included in Zhang *et al*. [[52]](#_ENREF_52)

**Table S11.** Genes with three or more recurrent mutations at the same position out of 40 exomes.

| Chr. | Position | SNV | Gene | # of samples mutated | | # of MSI samples mutated |
| --- | --- | --- | --- | --- | --- | --- |
| 2 | 148400156 | frameshift | ACVR2A | 4 | 4 | |
| 1 | 6180372 | frameshift | RPL22 | 3 | 3 | |
| 18 | 55164174 | frameshift | LMAN1 | 3 | 3 | |
| 3 | 155515672 | frameshift | DHX36 | 3 | 3 | |
| 8 | 74670025 | frameshift | STAU2 | 3 | 3 | |
| 10 | 27499062 | frameshift | MASTL | 3 | 3 | |
| 10 | 89707750 | frameshift | PTEN | 3 | 3 | |
| 14 | 57884204 | frameshift | ARID4A | 3 | 3 | |
| 1 | 198860665 | frameshift | DDX59 | 3 | 3 | |
| 6 | 46768374 | frameshift | TDRD6 | 3 | 2 | |
| 1 | 150436951 | frameshift | FLG2 | 3 | 1 | |
| 17 | 71529149 | missense | EVPL | 3 | 0 | |
| 9 | 33375815 | missense | AQP7 | 3 | 0 | |

**Table S13.** Genes recurrently mutated by non-synonymous SNVs or indels in *TP53*-wild-type GC samples (≥ 4 out of 20 exomes).

| Gene symbol | Name | Size | # of mutated  samples | # of mutated samples/ Length |
| --- | --- | --- | --- | --- |
| *PTEN* | phosphatidylinositol-3,4,5-trisphosphate | 1212 | 5 | 0.00413 |
| *ACVR2A* | activin receptor type-2A precursor | 1542 | 4 | 0.00259 |
| *TTK* | dual specificity protein kinase TTK isoform 1 | 2574 | 4 | 0.00155 |
| *ARID1A* | AT-rich interactive domain-containing protein 1A | 6858 | 6 | 0.00087 |
| *PCDH15* | protocadherin-15 isoform CD3-2 precursor | 5889 | 5 | 0.00085 |
| *PAPPA* | pregnancy-associated plasma protein A, pappalysin 1 | 4884 | 4 | 0.00082 |
| *DNAH7* | dynein heavy chain 7, axonemal | 12075 | 6 | 0.0005 |
| *DMD* | dystrophin Dp140c isoform | 11058 | 4 | 0.00036 |
| *LRP1B* | low-density lipoprotein receptor-related protein | 13800 | 4 | 0.00029 |
| *FAT4* | protocadherin Fat 4 precursor | 14946 | 4 | 0.00027 |
| *SYNE2* | nesprin-2 isoform 5 | 20724 | 4 | 0.00019 |

**Figure S1.** Mechanistic interpretation of major rearrangements of KRAS amplicon shown in Figure 1. A 1.9 Mbp deletion is followed by breakage-fusion-bridge (BFB) cycles. Schematic representation of chromosome 12 (green), with black circles representing centromeres. Gray arrows indicate the direction of increasing genomic coordinates and numbers indicate DNA-PET cluster sizes.

Figure S2. Amplification of a region on chromosome 6 in gastric tumor NGCII092 by BFB cycles. (a) Copy number profile of chromosome 6. *PAK1IP1* is located 290 kbp downstream of the sharp increase in copy number. (b) Rearrangements identified by DNA-PET clusters with size ≥50 are represented by arrows and connecting lines. Clusters are arranged according to size (number of PETs are shown for each rearrangement point). Dark red and pink arrows represent left and right anchors (tag mapping regions) of PET clusters with the connection between the tip of the dark red and the blunt end of the pink arrows. Unpaired inversions with a short distance between their breakpoints represented by dark red and pink arrows in different orientation and close proximity indicate head to head or tail to tail fusions of BFB cycles. (c) Interpretation of the DNA-PET data by BFB cycles. Chromosome 6 is represented by grey lines with black circles as centromeres. Orientations of genomic segments are indicated by gray arrows from small to large coordinates. Numbers correspond to DNA-PET cluster sizes in (b). Cycle 1 is likely to be the first rearrangement at this locus followed by a series of other cycles including different rearrangement types. The data implies the propagation of different populations of rearranged chromosomes which together result in the amplification.

**Figure S3:** Validation of fusion genes by PCR and Sanger sequencing. **(a)** Genomic PCR products of tumor (T) and blood (N, normal; M, marker) were separated by electrophoresis on a 1% agarose gel. Multiple bands in the normal sample for YWHAB-BCAS1 were not sequenced. OVCH1-CCDC91 amplicons in the normal sample were determined to be unspecific by Sanger sequencing. **(b)** RT-PCR reactions which resulted in amplicons from tumor samples are shown. **(c)** Sanger sequencing of genomic fusion points of the two expressed fusion genes in (b). **(d)** Sanger sequencing results of RT-PCR products of fusion genes in (b).

**Figure S4.** Complex rearrangement between *YWHAB, BCAS1* and *DOK5* with signature of a replication coupled rearrangement mechanism. **(a)** Genome Browser view shows coordinates of two genomic regions on chromosome 20 with UCSC known gene information (Hsu et al. 2006) (top) and copy number and rearrangement information for tumor and blood (middle). Color coding of relative genomic positions which correspond to the code in (b) is shown on the bottom. Rearrangements are indicated by dark red and pink arrows. PET cluster sizes are indicated by red numbers followed by strand and start and end coordinates of left and right mapping regions. **(b)** Reconstructed architecture of the tumor genome with hg18 genomic coordinates given on top and bottom. Orientations are indicated by arrow heads. Genomic fragment sizes are indicated in bold, micro-homology at fusion points is shown in italic. **(c)** Mapping regions of DNA-PET clusters relative to the reconstructed sequence in (b). 5’ and 3’ mapping regions are indicated by dark red and pink arrow heads. Red numbers indicate cluster sizes which correspond to (a). Breakpoints have been validated by Sanger sequencing of three PCR products for fragments A-D, D-F, and F-G respectively. The dashed line indicates that PETs of two different rearrangement points have been clustered together. Note that (b) and (c) are not drawn to scale.


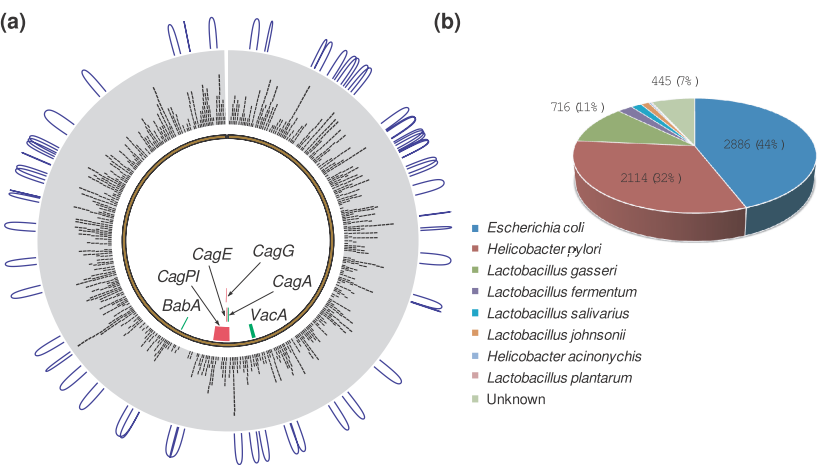
 Figure S5. Microbiome in the *H. pylori* infected tumor sample (NGCII082). (a) Circos plot depicting WGS read coverage (black bars in grey ring) and DNA-PET fragment coverage (blue loops outside grey ring) of the reference *H. pylori* shi470 genome. Location of key virulence genes (in green) and the Cag PI (in red) are marked by corresponding boxes in the inner circle (b) Frequency distribution of WGS reads associated with various bacterial species found in NGCII082.


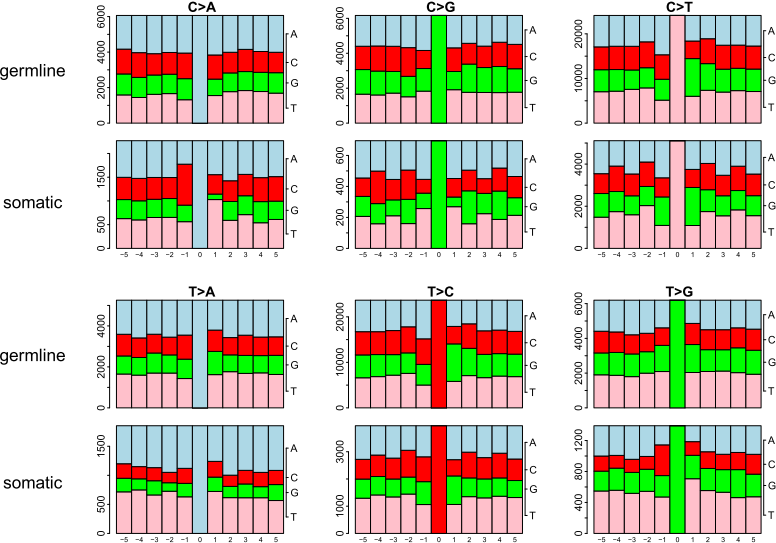


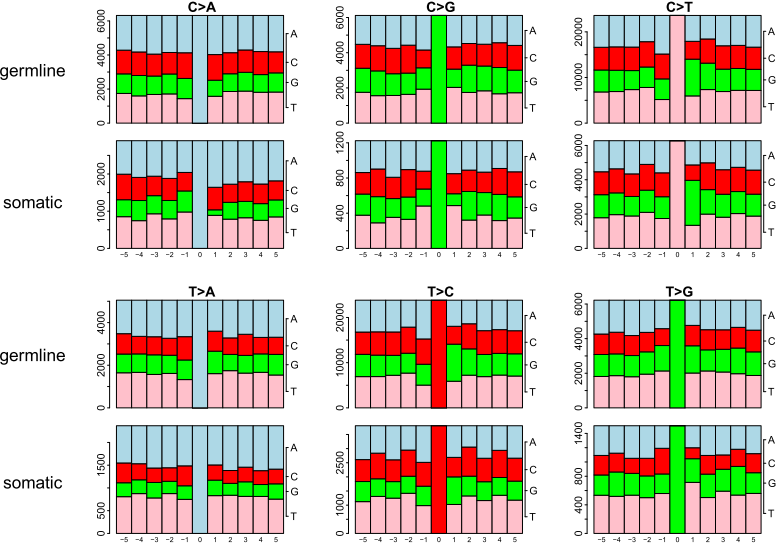


**(b)**

**(a)**

Figure S6. Frequency of bases adjacent to somatic SNVs in various mutational classes. (a) Sample NGCII082 (b) Sample NGCII092. Color codes represent nucleotides as indicated on the right of each panel. Scale for number of observations is given on the left side of each panel.

**
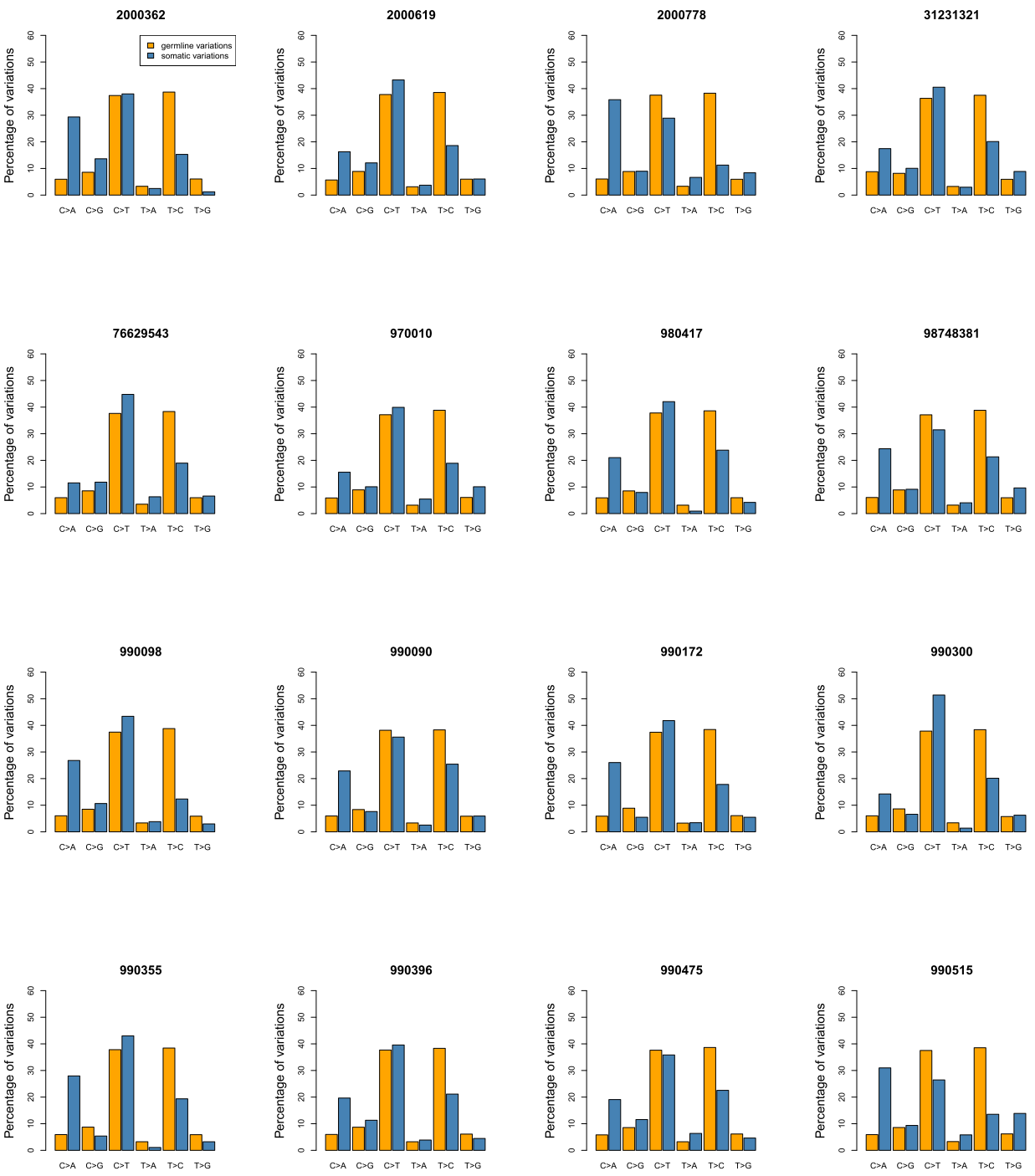
**

**Figure S7.** Mutational fingerprint in a set of 16 exome-sequenced tumors detailed in **Table S8**. Each graph shows the exome-wide frequency of germline and somatic SNVs in the corresponding tumor.


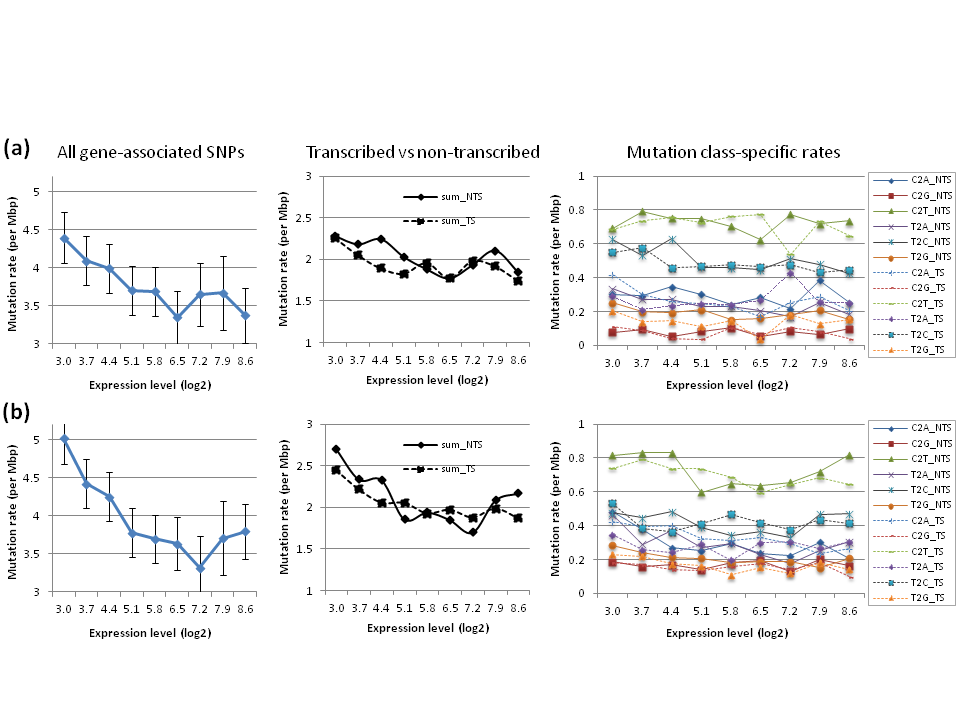


**Figure S8.** Mutation rate as a function of expression level. **(a)** Sample NGCII082 **(b)** Sample NGCII092. The abbreviations TS and NTS refer to SNVs on the transcribed and non-transcribed strand respectively. The values reported are averaged over all genes at a particular expression level.

**Figure S9**. The sequences flanking the A/T deletion for 2 Normal/Tumor sample pairs (TGCII069 and TGCII087) are shown for **(a)** *ACVR2A*, **(b)** *LMAN1*, and **(c)** *RPL22.* *ACVR2A* is located on the plus strand whereas *LMAN1* and *RPL22* are on the minus strand. For each sample pair, the sequence of the tumor sample is aligned below that of the normal sample. The vertical red line indicates the point where a shift in sequence is detected in the tumor samples due to a single A/T deletion in the homopolymer region upstream of the vertical red line. For *ACVR2A*, the tumor sample of TGCII087 shows the deletion of a single A/T with a frequency of 100%, suggesting a homozygous state (maybe due to loss of heterozygosity), whereas a heterozygous deletion in the TGCII069 tumor sample is indicated by the overlapping peaks in the sequence trace downstream of the vertical red line. For *LMAN1* and *RPL22*, both tumor samples showed heterozygous deletions.


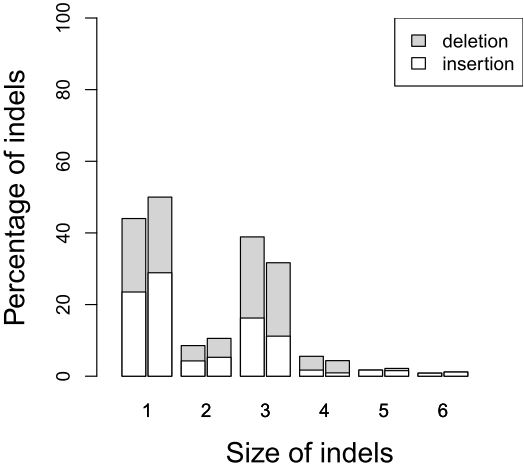


Figure S10. Size distribution of exome-wide germline indels. For each size of indels, data for NGCII082 is presented on the left bar and for NGCII092 on the right bar.

(a)

(b)

Figure S11. Gene expression analysis based on Affymetrix microarray U133 plus of 13 gastric tumors. (a) Expression of *PTEN* in gastric tumor NGCII082 compared to twelve other gastric tumors. Probe sets mapping to exons 1, 7 and 9 were analysed for log2 expression differences between gastric tumor of patient NGCII082 and 12 other gastric tumors. Error bars indicate standard deviation across twelve tumor samples. (b) Expression of *SMAD4* in gastric tumor NGCII092 compared to twelve other gastric tumors.


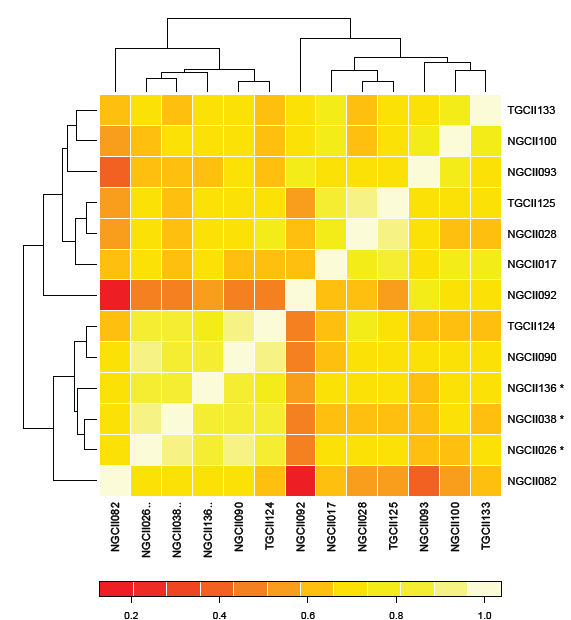


Figure S12. Gene expression clustering analysis of 13 gastric tumors. Expression values of 514 differentially expressed probe sets were extracted from the 13 tumors and clustered using the "heatmap_2" function of the Heatplus package in R. Patients known to have had tumors that metastasized are marked with an asterisk. Colour code represents correlation of expression values over the 514 probe sets.


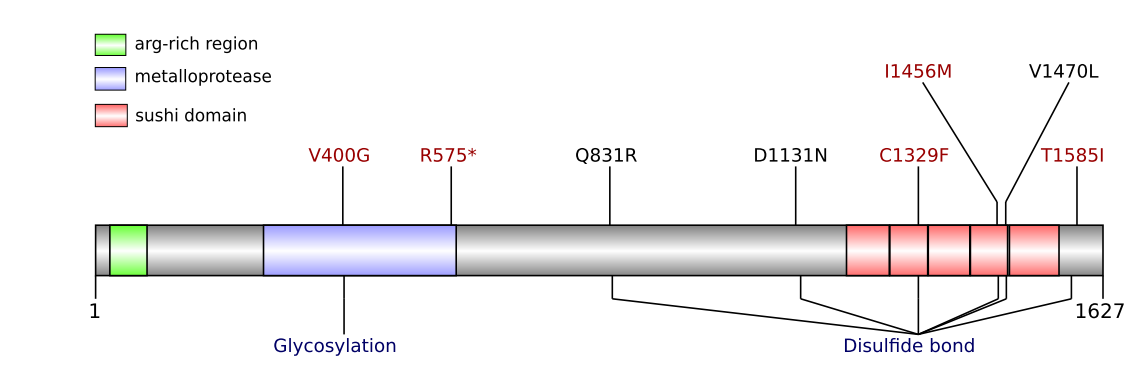


Figure S13. *PAPPA* mutations identified in whole-genome, exome and targeted sequencing data. Non-synonymous SNVs (those marked deleterious by SIFT in red) are shown above the protein (coordinates in aa) and protein domain and functional site information was obtained from UniProt (<http://www.uniprot.org/>). The *PAPPA* gene is mutated in other cancers as well (http://cancer.sanger.ac.uk/cancergenome/projects/cosmic/), shows weak but detectable expression in most gastric tumors (Figure S14) and many of the mutations observed here occur close to functional sites in this multi-domain protein.

**(b)**

**(a)**

Figure S14. Expression analysis of *PAPPA* by quantitative PCR (qPCR) of 14 gastric tumors and three gastric cell lines. One microgram RNA of each of fourteen gastric tumors and the three gastric cell lines TMK1, HGC27, and AZ521 has been reverse transcribed using Superscript III (Life Technologies) in a 21 µl reaction volume. One microliter has been used for qPCR using SybrGreen (Life Technologies) in a LightCycler 480 device (Roche) in a 384 well format with the following primers: PAPPA_RT_F1, TGGCGATGGCATTATACAAA and PAPPA_RT_R1, CACATACCCCATCACCATCA. (a) Raw Cycle thresholds (Ct) for each sample are shown. (b) *GAPDH* has been used as housekeeping control for normalization allowing sample to sample comparison by ΔCt values. Error bars indicate standard deviation of triplicates.

References

1. Kidd JM, Cooper GM, Donahue WF, Hayden HS, Sampas N, Graves T, Hansen N, Teague B, Alkan C, Antonacci F, Haugen E, Zerr T, Yamada NA, Tsang P, Newman TL, Tuzun E, Cheng Z, Ebling HM, Tusneem N, David R, Gillett W, Phelps KA, Weaver M, Saranga D, Brand A, Tao W, Gustafson E, McKernan K, Chen L, Malig M *et al*: Mapping and sequencing of structural variation from eight human genomes. *Nature* 2008, 453:56-64.

2. Korbel JO, Urban AE, Affourtit JP, Godwin B, Grubert F, Simons JF, Kim PM, Palejev D, Carriero NJ, Du L, Taillon BE, Chen Z, Tanzer A, Saunders AC, Chi J, Yang F, Carter NP, Hurles ME, Weissman SM, Harkins TT, Gerstein MB, Egholm M, Snyder M: Paired-end mapping reveals extensive structural variation in the human genome. *Science* 2007, 318:420-426.

3. Chen K, Wallis JW, McLellan MD, Larson DE, Kalicki JM, Pohl CS, McGrath SD, Wendl MC, Zhang Q, Locke DP, Shi X, Fulton RS, Ley TJ, Wilson RK, Ding L, Mardis ER: BreakDancer: an algorithm for high-resolution mapping of genomic structural variation. *Nat Methods* 2009, 6:677-681.

4. Hillmer AM, Yao F, Inaki K, Lee WH, Ariyaratne PN, Teo AS, Woo XY, Zhang Z, Zhao H, Ukil L, Chen JP, Zhu F, So JB, Salto-Tellez M, Poh WT, Zawack KF, Nagarajan N, Gao S, Li G, Kumar V, Lim HP, Sia YY, Chan CS, Leong ST, Neo SC, Choi PS, Thoreau H, Tan PB, Shahab A, Ruan X *et al*: Comprehensive long-span paired-end-tag mapping reveals characteristic patterns of structural variations in epithelial cancer genomes. *Genome Res* 2011, 21:665-675.

5. Lock FE, Underhill-Day N, Dunwell T, Matallanas D, Cooper W, Hesson L, Recino A, Ward A, Pavlova T, Zabarovsky E, Grant MM, Maher ER, Chalmers AD, Kolch W, Latif F: The RASSF8 candidate tumor suppressor inhibits cell growth and regulates the Wnt and NF-kappaB signaling pathways. *Oncogene*, 29:4307-4316.

6. Deng N, Goh LK, Wang H, Das K, Tao J, Tan IB, Zhang S, Lee M, Wu J, Lim KH, Lei Z, Goh G, Lim QY, Lay-Keng Tan A, Sin Poh DY, Riahi S, Bell S, Shi MM, Linnartz R, Zhu F, Yeoh KG, Toh HC, Yong WP, Cheong HC, Rha SY, Boussioutas A, Grabsch H, Rozen S, Tan P: A comprehensive survey of genomic alterations in gastric cancer reveals systematic patterns of molecular exclusivity and co-occurrence among distinct therapeutic targets. *Gut* 2012.

7. Xia C, Ma W, Stafford LJ, Marcus S, Xiong WC, Liu M: Regulation of the p21-activated kinase (PAK) by a human Gbeta -like WD-repeat protein, hPIP1. *Proceedings of the National Academy of Sciences of the United States of America* 2001, 98:6174-6179.

8. Gu W, Zhang F, Lupski JR: Mechanisms for human genomic rearrangements. *Pathogenetics* 2008, 1:4.

9. De S, Michor F: DNA replication timing and long-range DNA interactions predict mutational landscapes of cancer genomes. *Nat Biotechnol* 2011, 29:1103-1108.

10. Berger MF, Lawrence MS, Demichelis F, Drier Y, Cibulskis K, Sivachenko AY, Sboner A, Esgueva R, Pflueger D, Sougnez C, Onofrio R, Carter SL, Park K, Habegger L, Ambrogio L, Fennell T, Parkin M, Saksena G, Voet D, Ramos AH, Pugh TJ, Wilkinson J, Fisher S, Winckler W, Mahan S, Ardlie K, Baldwin J, Simons JW, Kitabayashi N, MacDonald TY *et al*: The genomic complexity of primary human prostate cancer. *Nature* 2011, 470:214-220.

11. Ding L, Ellis MJ, Li S, Larson DE, Chen K, Wallis JW, Harris CC, McLellan MD, Fulton RS, Fulton LL, Abbott RM, Hoog J, Dooling DJ, Koboldt DC, Schmidt H, Kalicki J, Zhang Q, Chen L, Lin L, Wendl MC, McMichael JF, Magrini VJ, Cook L, McGrath SD, Vickery TL, Appelbaum E, Deschryver K, Davies S, Guintoli T, Crowder R *et al*: Genome remodelling in a basal-like breast cancer metastasis and xenograft. *Nature* 2010, 464:999-1005.

12. Lee W, Jiang Z, Liu J, Haverty PM, Guan Y, Stinson J, Yue P, Zhang Y, Pant KP, Bhatt D, Ha C, Johnson S, Kennemer MI, Mohan S, Nazarenko I, Watanabe C, Sparks AB, Shames DS, Gentleman R, de Sauvage FJ, Stern H, Pandita A, Ballinger DG, Drmanac R, Modrusan Z, Seshagiri S, Zhang Z: The mutation spectrum revealed by paired genome sequences from a lung cancer patient. *Nature* 2010, 465:473-477.

13. Ley TJ, Mardis ER, Ding L, Fulton B, McLellan MD, Chen K, Dooling D, Dunford-Shore BH, McGrath S, Hickenbotham M, Cook L, Abbott R, Larson DE, Koboldt DC, Pohl C, Smith S, Hawkins A, Abbott S, Locke D, Hillier LW, Miner T, Fulton L, Magrini V, Wylie T, Glasscock J, Conyers J, Sander N, Shi X, Osborne JR, Minx P *et al*: DNA sequencing of a cytogenetically normal acute myeloid leukaemia genome. *Nature* 2008, 456:66-72.

14. Shah SP, Morin RD, Khattra J, Prentice L, Pugh T, Burleigh A, Delaney A, Gelmon K, Guliany R, Senz J, Steidl C, Holt RA, Jones S, Sun M, Leung G, Moore R, Severson T, Taylor GA, Teschendorff AE, Tse K, Turashvili G, Varhol R, Warren RL, Watson P, Zhao Y, Caldas C, Huntsman D, Hirst M, Marra MA, Aparicio S: Mutational evolution in a lobular breast tumour profiled at single nucleotide resolution. *Nature* 2009, 461:809-813.

15. Rieder G, Merchant JL, Haas R: Helicobacter pylori cag-type IV secretion system facilitates corpus colonization to induce precancerous conditions in Mongolian gerbils. *Gastroenterology* 2005, 128:1229-1242.

16. Tegtmeyer N, Wessler S, Backert S: Role of the cag-pathogenicity island encoded type IV secretion system in Helicobacter pylori pathogenesis. *FEBS J* 2011, 278:1190-1202.

17. Glocker E, Lange C, Covacci A, Bereswill S, Kist M, Pahl HL: Proteins encoded by the cag pathogenicity island of Helicobacter pylori are required for NF-kappaB activation. *Infect Immun* 1998, 66:2346-2348.

18. Sharma SA, Tummuru MK, Miller GG, Blaser MJ: Interleukin-8 response of gastric epithelial cell lines to Helicobacter pylori stimulation in vitro. *Infect Immun* 1995, 63:1681-1687.

19. van Doorn LJ, Figueiredo C, Sanna R, Plaisier A, Schneeberger P, de Boer W, Quint W: Clinical relevance of the cagA, vacA, and iceA status of Helicobacter pylori. *Gastroenterology* 1998, 115:58-66.

20. Gerhard M, Lehn N, Neumayer N, Boren T, Rad R, Schepp W, Miehlke S, Classen M, Prinz C: Clinical relevance of the Helicobacter pylori gene for blood-group antigen-binding adhesin. *Proceedings of the National Academy of Sciences of the United States of America* 1999, 96:12778-12783.

21. Peek RM, Jr., Blaser MJ: Helicobacter pylori and gastrointestinal tract adenocarcinomas. *Nature reviews Cancer* 2002, 2:28-37.

22. Schubert ML, Peura DA: Control of gastric acid secretion in health and disease. *Gastroenterology* 2008, 134:1842-1860.

23. Bik EM, Eckburg PB, Gill SR, Nelson KE, Purdom EA, Francois F, Perez-Perez G, Blaser MJ, Relman DA: Molecular analysis of the bacterial microbiota in the human stomach. *Proc Natl Acad Sci U S A* 2006, 103:732-737.

24. Aiba Y, Suzuki N, Kabir AM, Takagi A, Koga Y: Lactic acid-mediated suppression of Helicobacter pylori by the oral administration of Lactobacillus salivarius as a probiotic in a gnotobiotic murine model. *Am J Gastroenterol* 1998, 93:2097-2101.

25. Johnson-Henry KC, Mitchell DJ, Avitzur Y, Galindo-Mata E, Jones NL, Sherman PM: Probiotics reduce bacterial colonization and gastric inflammation in H. pylori-infected mice. *Dig Dis Sci* 2004, 49:1095-1102.

26. Totoki Y, Tatsuno K, Yamamoto S, Arai Y, Hosoda F, Ishikawa S, Tsutsumi S, Sonoda K, Totsuka H, Shirakihara T, Sakamoto H, Wang L, Ojima H, Shimada K, Kosuge T, Okusaka T, Kato K, Kusuda J, Yoshida T, Aburatani H, Shibata T: High-resolution characterization of a hepatocellular carcinoma genome. *Nat Genet* 2011, 43:464-469.

27. Pleasance ED, Cheetham RK, Stephens PJ, McBride DJ, Humphray SJ, Greenman CD, Varela I, Lin ML, Ordonez GR, Bignell GR, Ye K, Alipaz J, Bauer MJ, Beare D, Butler A, Carter RJ, Chen L, Cox AJ, Edkins S, Kokko-Gonzales PI, Gormley NA, Grocock RJ, Haudenschild CD, Hims MM, James T, Jia M, Kingsbury Z, Leroy C, Marshall J, Menzies A *et al*: A comprehensive catalogue of somatic mutations from a human cancer genome. *Nature* 2010, 463:191-196.

28. Pleasance ED, Stephens PJ, O'Meara S, McBride DJ, Meynert A, Jones D, Lin ML, Beare D, Lau KW, Greenman C, Varela I, Nik-Zainal S, Davies HR, Ordonez GR, Mudie LJ, Latimer C, Edkins S, Stebbings L, Chen L, Jia M, Leroy C, Marshall J, Menzies A, Butler A, Teague JW, Mangion J, Sun YA, McLaughlin SF, Peckham HE, Tsung EF *et al*: A small-cell lung cancer genome with complex signatures of tobacco exposure. *Nature* 2010, 463:184-190.

29. Lengauer C, Kinzler KW, Vogelstein B: Genetic instabilities in human cancers. *Nature* 1998, 396:643-649.

30. Stenson PD, Mort M, Ball EV, Howells K, Phillips AD, Thomas NS, Cooper DN: The Human Gene Mutation Database: 2008 update. *Genome Med* 2009, 1:13.

31. Wang H, Zhou Y, Zhuang W, Yin YQ, Liu GJ, Wu TX, Yao X, Du L, Wei ML, Wu XT: Glutathione S-transferase M1 null genotype associated with gastric cancer among Asians. *Dig Dis Sci* 2010, 55:1824-1830.

32. Lunn RM, Langlois RG, Hsieh LL, Thompson CL, Bell DA: XRCC1 polymorphisms: effects on aflatoxin B1-DNA adducts and glycophorin A variant frequency. *Cancer Res* 1999, 59:2557-2561.

33. Takanami T, Nakamura J, Kubota Y, Horiuchi S: The Arg280His polymorphism in X-ray repair cross-complementing gene 1 impairs DNA repair ability. *Mutat Res* 2005, 582:135-145.

34. Ju H, Lim B, Kim M, Noh SM, Han DS, Yu HJ, Choi BY, Kim YS, Kim WH, Ihm C, Kang C: Genetic variants A1826H and D2937Y in GAG-beta domain of versican influence susceptibility to intestinal-type gastric cancer. *J Cancer Res Clin Oncol* 2010, 136:195-201.

35. Tommasi S, Fedele V, Lacalamita R, Bruno M, Schittulli F, Ginzinger D, Scott G, Eppenberger-Castori S, Calistri D, Casadei S, Seymour I, Longo S, Giannelli G, Pilato B, Simone G, Benz CC, Paradiso A: 655Val and 1170Pro ERBB2 SNPs in familial breast cancer risk and BRCA1 alterations. *Cell Oncol* 2007, 29:241-248.

36. Shin YK, Heo SC, Shin JH, Hong SH, Ku JL, Yoo BC, Kim IJ, Park JG: Germline mutations in MLH1, MSH2 and MSH6 in Korean hereditary non-polyposis colorectal cancer families. *Hum Mutat* 2004, 24:351.

37. Li W, Li JF, Qu Y, Chen XH, Qin JM, Gu QL, Yan M, Zhu ZG, Liu BY: Comparative proteomics analysis of human gastric cancer. *World J Gastroenterol* 2008, 14:5657-5664.

38. Park WS, Lee JH, Shin MS, Park JY, Kim HS, Kim YS, Lee SN, Xiao W, Park CH, Lee SH, Yoo NJ, Lee JY: Inactivating mutations of the caspase-10 gene in gastric cancer. *Oncogene* 2002, 21:2919-2925.

39. Wang LH, Kim SH, Lee JH, Choi YL, Kim YC, Park TS, Hong YC, Wu CF, Shin YK: Inactivation of SMAD4 tumor suppressor gene during gastric carcinoma progression. *Clin Cancer Res* 2007, 13:102-110.

40. Mack JT, Brown CB, Tew KD: ABCA2 as a therapeutic target in cancer and nervous system disorders. *Expert Opin Ther Targets* 2008, 12:491-504.

41. Jurica MS, Licklider LJ, Gygi SR, Grigorieff N, Moore MJ: Purification and characterization of native spliceosomes suitable for three-dimensional structural analysis. *RNA* 2002, 8:426-439.

42. Futterer A, Campanero MR, Leonardo E, Criado LM, Flores JM, Hernandez JM, San Miguel JF, Martinez AC: Dido gene expression alterations are implicated in the induction of hematological myeloid neoplasms. *J Clin Invest* 2005, 115:2351-2362.

43. Clark J, Lu YJ, Sidhar SK, Parker C, Gill S, Smedley D, Hamoudi R, Linehan WM, Shipley J, Cooper CS: Fusion of splicing factor genes PSF and NonO (p54nrb) to the TFE3 gene in papillary renal cell carcinoma. *Oncogene* 1997, 15:2233-2239.

44. Fousteri M, Vermeulen W, van Zeeland AA, Mullenders LH: Cockayne syndrome A and B proteins differentially regulate recruitment of chromatin remodeling and repair factors to stalled RNA polymerase II in vivo. *Mol Cell* 2006, 23:471-482.

45. Makiniemi M, Hillukkala T, Tuusa J, Reini K, Vaara M, Huang D, Pospiech H, Majuri I, Westerling T, Makela TP, Syvaoja JE: BRCT domain-containing protein TopBP1 functions in DNA replication and damage response. *J Biol Chem* 2001, 276:30399-30406.

46. Ng PC, Henikoff S: SIFT: Predicting amino acid changes that affect protein function. *Nucleic Acids Res* 2003, 31:3812-3814.

47. Kaminker JS, Zhang Y, Watanabe C, Zhang Z: CanPredict: a computational tool for predicting cancer-associated missense mutations. *Nucleic Acids Res* 2007, 35:W595-598.

48. Adzhubei IA, Schmidt S, Peshkin L, Ramensky VE, Gerasimova A, Bork P, Kondrashov AS, Sunyaev SR: A method and server for predicting damaging missense mutations. *Nat Methods* 2010, 7:248-249.

49. Qi Q, Zhao Y, Li M, Simon R: Non-negative matrix factorization of gene expression profiles: a plug-in for BRB-ArrayTools. *Bioinformatics* 2009, 25:545-547.

50. Vecchi M, Nuciforo P, Romagnoli S, Confalonieri S, Pellegrini C, Serio G, Quarto M, Capra M, Roviaro GC, Contessini Avesani E, Corsi C, Coggi G, Di Fiore PP, Bosari S: Gene expression analysis of early and advanced gastric cancers. *Oncogene* 2007, 26:4284-4294.

51. Fock KM, Ang TL: Epidemiology of Helicobacter pylori infection and gastric cancer in Asia. *J Gastroenterol Hepatol* 2010, 25:479-486.

52. Zang ZJ, Cutcutache I, Poon SL, Zhang SL, McPherson JR, Tao J, Rajasegaran V, Heng HL, Deng N, Gan A, Lim KH, Ong CK, Huang D, Chin SY, Tan IB, Ng CC, Yu W, Wu Y, Lee M, Wu J, Poh D, Wan WK, Rha SY, So J, Salto-Tellez M, Yeoh KG, Wong WK, Zhu YJ, Futreal PA, Pang B *et al*: Exome sequencing of gastric adenocarcinoma identifies recurrent somatic mutations in cell adhesion and chromatin remodeling genes. *Nat Genet* 2012.
